# Supplementary material for: The homogenization of avian morphological and phylogenetic diversity under the global extinction crisis
Source: Curr Biol. 2022 Sep 12;32(17):3830–3837.e3. doi: 10.1016/j.cub.2022.06.018 (PMC9616725; doi:10.1016/j.cub.2022.06.018)
Supplement: Document S1. Figures S1–S4 and Tables S1 and S2 [file mmc1.pdf]

Current Biology, Volume *32*

Supplemental Information

The homogenization of avian morphological and phylogenetic diversity under the global extinction crisis

Emma C. Hughes, David P. Edwards, and Gavin H. Thomas

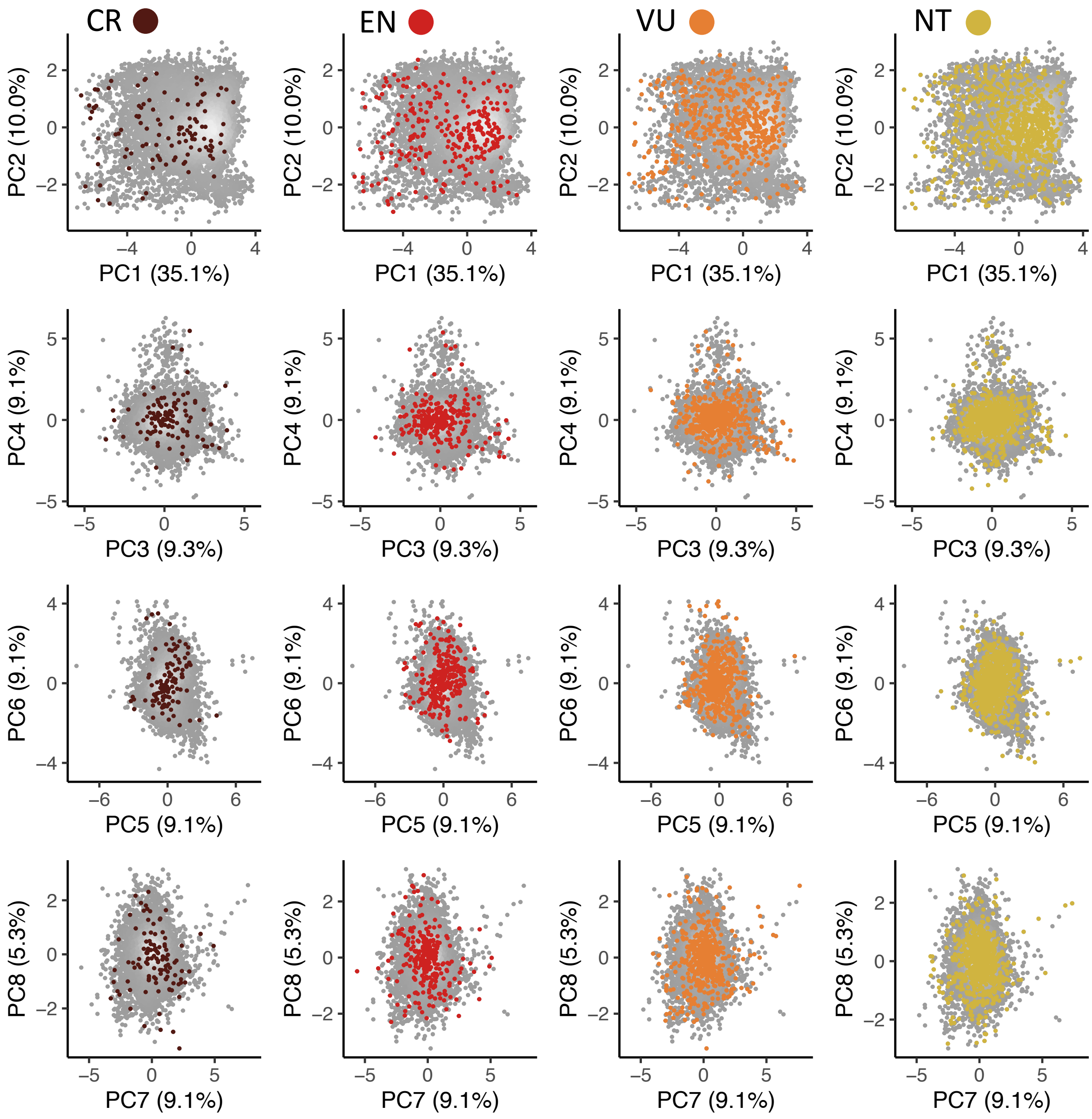

Figure S1: Scatterplots showing the first eight principal components of morphological traits, and the proportion of variation represented by each for each IUCN threat category. Related to Figure 1 and Table S1.

Species classified in the IUCN red list as Critically Endangered (CR), Endangered (EN), Vulnerable (VU), and Near Threatened (NT) are coloured dark red through to yellow, whilst all other species are grey. Light grey shows where species density is highest. The phylogenetic signal (mean λ) for each principal component is as follows: PC1 = 0.962, PC2 = 0.956, PC3 = 0.848, PC4 = 0.885, PC5 = 0.908, PC6 = 0.856, PC7 = 0.873, PC8 = 0.900.

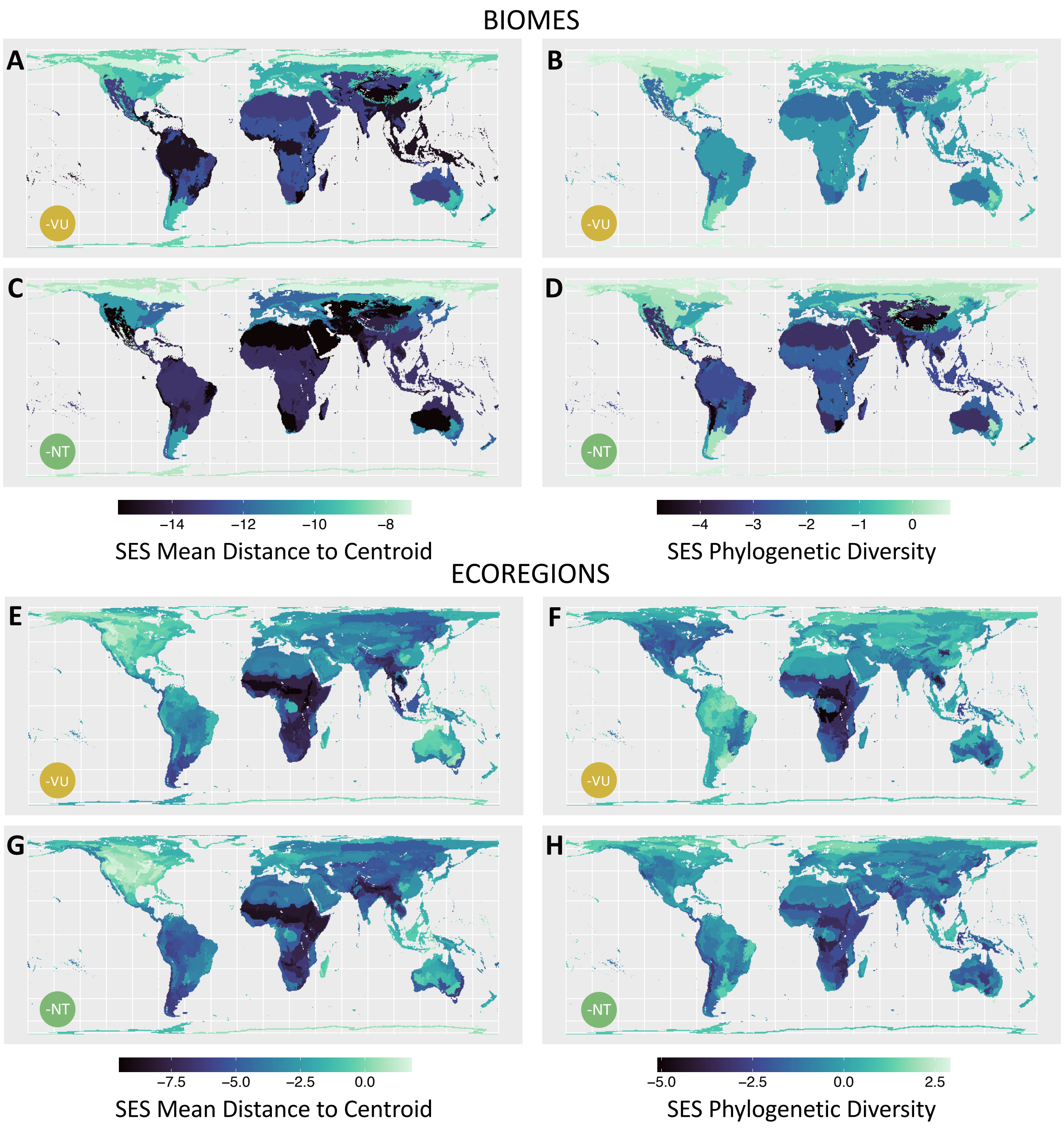

Figure S2: **Variation in morphological diversity and phylogenetic diversity across avian assemblages in each terrestrial biome and ecoregion. Related to Figures 3, 4, S3 and S4.**

Standard effect sizes (SES) for **A**) morphological and **B**) phylogenetic diversity were calculated after Critically Endangered, Endangered and Vulnerable species (-VU), and (**C, D**) additionally, when Near Threatened species (-NT) are dropped from 1000 simulated communities across 14 terrestrial biomes, and respectively across 814 terrestrial ecoregions (**E-H**). The darkest blue colour indicates where SES values are more negative than expected, with values < -2 showing significant homogenisation.

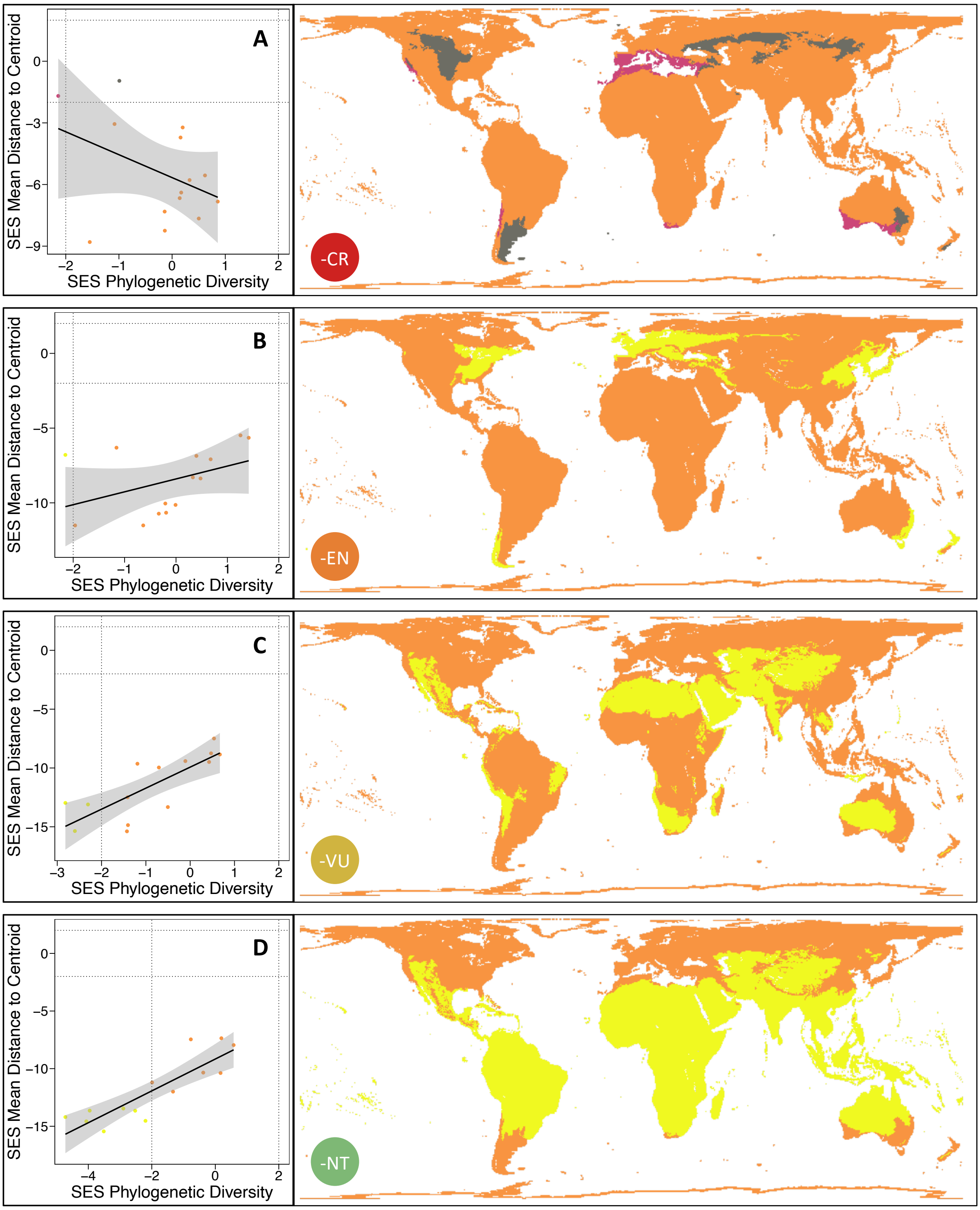

Figure S3: **Significant deviations from expected morphological and phylogenetic diversity across each terrestrial biome. Related to Figures 3 and S2.**
Standard effect sizes (SES) of morphological diversity (mean distance to centroid) and phylogenetic diversity of species assemblages in each global terrestrial biome (n = 14) where significant deviation from expected (+/- 2) is present. Homogenisation is indicated where SES is more negative than -2. Significant SES scores calculated when assemblages are missing **A)** Critically Endangered (-CR), plus **B)** Endangered (-EN), plus **C)** Vulnerable (-VU), plus **D)** Near Threatened (-NT) species. Dark grey indicates no significant deviation from expected.

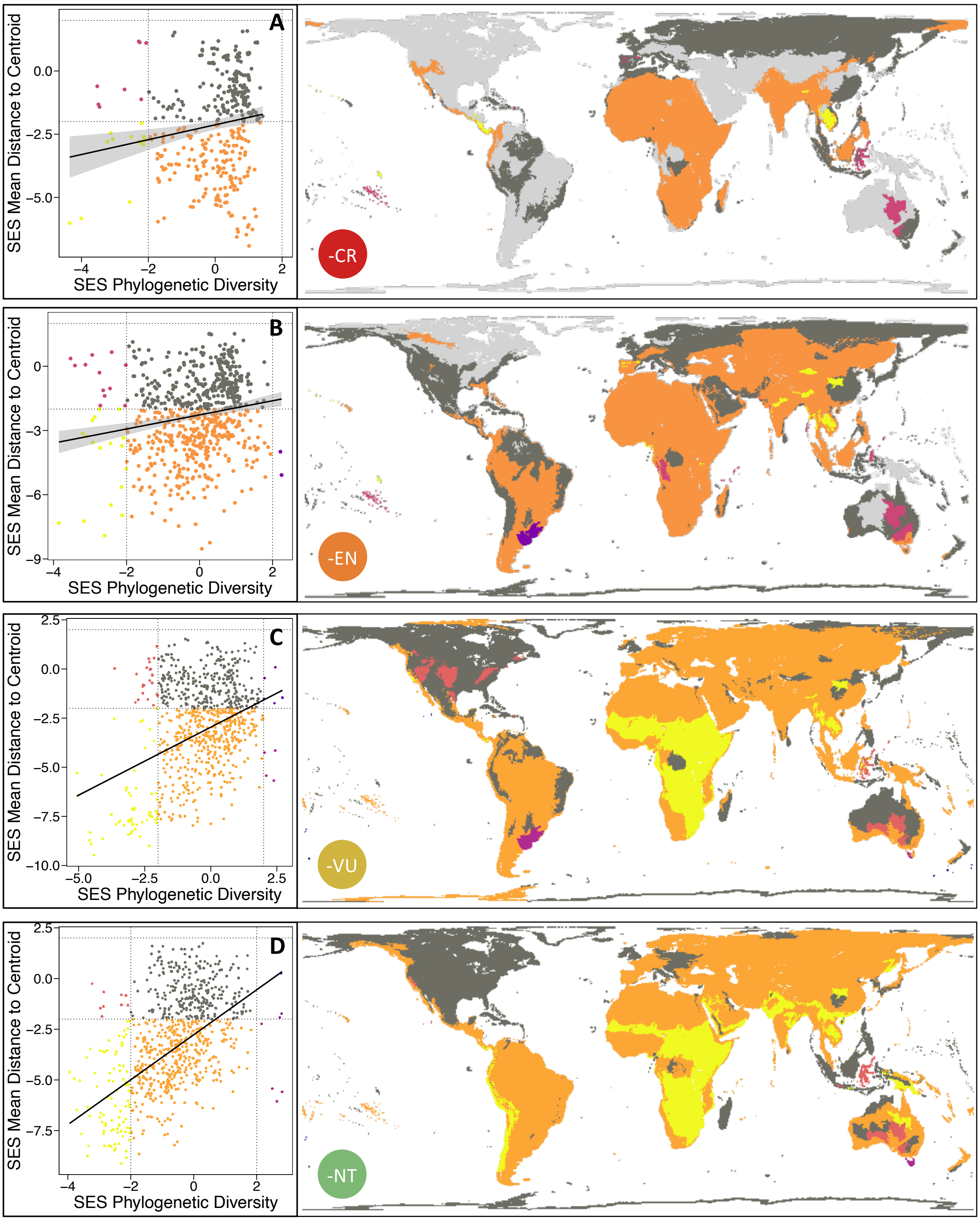

Figure S4: **Significant deviations from expected morphological and phylogenetic diversity across each terrestrial ecoregion. Related to Figures 4 and S2.**

Standard effect sizes (SES) of morphological diversity (mean distance to centroid) and phylogenetic diversity of species assemblages in each global terrestrial ecoregion (n = 814) where significant deviation from expected (+/- 2) is present. Homogenisation is indicated where SES is more negative than -2. Significant SES scores calculated when assemblages are missing **A)** Critically Endangered (-CR), plus **B)** Endangered (-EN), plus **C)** Vulnerable (-VU), plus **D)** Near Threatened (-NT) species. Dark grey indicates no significant deviation from expected. Light grey indicates ecoregions with no CR or EN species.

Table S1: Species richness, mean distance to centroid and the mean, standard deviation of null simulations used to calculate the SES. Related to Figure 1 and S1.

	Species richness	Mean distance to centroid	Null mean distance to centroid	Null SD distance to centroid	SES mean distance to centroid
**PC1**					
All species retained	8455	1.572	NA	NA	0
CR lost	8344	1.559	1.572	0.002	-7.732
EN lost	8070	1.533	1.572	0.003	-12.387
VU lost	7543	1.488	1.572	0.005	-16.320
NT lost (LC only retained)	6731	1.455	1.572	0.007	-15.768
**PC2**					
All species retained	8455	0.822	NA	NA	0
CR lost	8344	0.821	0.822	0.001	-0.323
EN lost	8070	0.819	0.822	0.001	-1.350
VU lost	7543	0.818	0.822	0.002	-1.373
NT lost (LC only retained)	6731	0.813	0.821	0.004	-2.275
**PC3**					
All species retained	8455	0.771	NA	NA	0
CR lost	8344	0.768	0.771	0.001	-3.541
EN lost	8070	0.760	0.771	0.002	-6.585
VU lost	7543	0.745	0.771	0.003	-9.920
NT lost (LC only retained)	6731	0.734	0.771	0.004	-10.443
**PC4**					
All species retained	8455	0.705	NA	NA	0
CR lost	8344	0.702	0.705	0.001	-2.480
EN lost	8070	0.702	0.705	0.002	-1.786
VU lost	7543	0.695	0.705	0.003	-3.724
NT lost (LC only retained)	6731	0.693	0.705	0.004	-2.997
**PC5**					
All species retained	8455	0.754	NA	NA	0
CR lost	8344	0.752	0.754	0.001	-1.961
EN lost	8070	0.747	0.754	0.002	-4.173
VU lost	7543	0.740	0.754	0.002	-5.815
NT lost (LC only retained)	6731	0.726	0.753	0.004	-7.703
**PC6**					
All species retained	8455	0.775	NA	NA	0
CR lost	8344	0.773	0.775	0.001	-2.634
EN lost	8070	0.770	0.775	0.002	-3.445

VU lost	7543	0.763	0.775	0.002	-5.083
NT lost (LC only retained)	6731	0.761	0.775	0.004	-4.007
**PC7**					
All species retained	8455	0.714	NA	NA	0
CR lost	8344	0.709	0.714	0.001	-5.419
EN lost	8070	0.702	0.714	0.002	-7.072
VU lost	7543	0.687	0.714	0.003	-10.732
NT lost (LC only retained)	6731	0.677	0.714	0.004	-9.615
**PC8**					
All species retained	8455	0.580	NA	NA	0
CR lost	8344	0.576	0.580	0.001	-5.061
EN lost	8070	0.573	0.580	0.001	-5.514
VU lost	7543	0.565	0.579	0.002	-7.317
NT lost (LC only retained)	6731	0.560	0.579	0.003	-6.721
**PC1-8**					
All species retained	8455	2.949	NA	NA	0
CR lost	8344	2.934	2.949	0.002	-7.887
EN lost	8070	2.906	2.949	0.004	-12.004
VU lost	7543	2.855	2.949	0.006	-15.937
NT lost (LC only retained)	6731	2.816	2.948	0.008	-15.796
**PC2-8**					
All species retained	8455	2.339	NA	NA	0
CR lost	8344	2.332	2.340	0.001	-5.243
EN lost	8070	2.318	2.340	0.003	-7.611
VU lost	7543	2.291	2.340	0.004	-11.333
NT lost (LC only retained)	6731	2.270	2.339	0.006	-10.681

Species richness, mean distance to centroid values (the average across all individual species Euclidean distance to the centroid of morphospace where Critically Endangered (CR), Endangered (EN), Vulnerable (VU) and Near Threatened (NT) species are sequentially lost), null mean distance to centroid (the average across all individual species in a simulated community where CR, EN, VU and NT species are sequentially lost), null standard deviation (SD) of the distance to centroid values (for each simulated community), and the standard effect size (SES) mean distance to centroid value ((mean distance to centroid – null mean distance to centroid)/null SD distance to centroid). SES values are significant +/- 2. Each null value was calculated from 1000 simulated communities. These calculations were repeated individually for PC1, PC2, PC3, PC4, PC5, PC6, PC7 and PC8, across all PCs (1-8), and PCs 2-8.

Table S2: Loadings for each individual trait on each principal component. Related to Figure S1 and Star Methods.

	PC1	PC2	PC3	PC4	PC5	PC6	PC7	PC8
Beak PC1	0.002	0.936	0.014	0.028	0.113	0.074	0.056	0.006
Beak PC2	-0.046	-0.084	0.225	0.274	0.163	0.901	-0.100	-0.104
Beak PC3	0.280	-0.019	0.306	0.461	0.143	-0.156	-0.142	0.739
Beak PC4	0.100	-0.044	0.450	-0.674	0.199	0.137	0.440	0.270
Beak PC5	-0.183	-0.046	0.476	0.354	0.435	-0.353	0.274	-0.442
Beak PC6	-0.044	0.083	0.532	0.088	-0.824	-0.018	0.034	-0.085
Beak PC7	0.051	-0.041	-0.337	0.344	-0.178	0.121	0.835	0.119
Centroid Size	-0.451	-0.279	0.026	0.006	<0.001	<0.001	<0.001	0.139
Body Mass	-0.493	0.113	0.006	0.053	<0.001	<0.001	<0.001	0.123
Tarsus Length	-0.438	0.043	-0.144	-0.026	<0.001	<0.001	<0.001	0.316
Wing Length	-0.483	0.103	0.087	-0.039	<0.001	<0.001	<0.001	0.140
