# Supplementary material for: The homogenization of avian morphological and phylogenetic diversity under the global extinction crisis
Source: Curr Biol. 2022 Sep 12;32(17):3830–3837.e3. doi: 10.1016/j.cub.2022.06.018 (PMC9616725; doi:10.1016/j.cub.2022.06.018)
Supplement: Document S2. Article plus supplemental information [file mmc2.pdf]

Report

Current Biology

The homogenization of avian morphological and phylogenetic diversity under the global extinction crisis

Highlights

- Predicted loss of birds will drive exceptional declines in morphological diversity
- Species extinctions lead to a major loss of ecological strategies and functions
- Most biomes and ecoregions will experience morphological homogenization
- Phylogenetic diversity tends to decline as expected as species go extinct

Authors

Emma C. Hughes, David P. Edwards, Gavin H. Thomas

Correspondence

echughes8@gmail.com (E.C.H.), gavin.thomas@sheffield.ac.uk (G.H.T.)

In brief

The global extinction crisis will lead to widespread losses of morphological diversity. Hughes et al. show that predicted species extinctions drive far greater declines of ecological strategies than predicted, with important ramifications for humans as ecosystem services are lost. In contrast, phylogenetic diversity declines as expected.

Hughes et al., 2022, Current Biology *32*, 3830–3837
September 12, 2022 © 2022 The Author(s). Published by Elsevier Inc.
https://doi.org/10.1016/j.cub.2022.06.018

CellPress

CellPress
OPEN ACCESS

Current Biology

Report

The homogenization of avian morphological and phylogenetic diversity under the global extinction crisis

Emma C. Hughes,[1,2,3,4,*] David P. Edwards,[1] and Gavin H. Thomas[1,2,*]
[1]Ecology and Evolutionary Biology, School of Biosciences, University of Sheffield, Sheffield S10 2TN, UK
[2]Bird Group, Department of Life Sciences, Natural History Museum, Akeman Street, Tring HP23 6AP, UK
[3]Twitter handle: @EHughes27
[4]Lead contact
*Correspondence: echughes8@gmail.com (E.C.H.), gavin.thomas@sheffield.ac.uk (G.H.T.)
https://doi.org/10.1016/j.cub.2022.06.018

SUMMARY

Biodiversity is facing a global extinction crisis that will reduce ecological trait diversity, evolutionary history, and ultimately ecosystem functioning and services.[1–4] A key challenge is understanding how species losses will impact morphological and phylogenetic diversity at global scales.[5,6] Here, we test whether the loss of species threatened with extinction according to the International Union for Conservation of Nature (IUCN) leads to morphological and phylogenetic homogenization[7,8] across both the whole avian class and within each biome and ecoregion globally. We use a comprehensive set of continuous morphological traits extracted from museum collections of 8,455 bird species, including geometric morphometric beak shape data,[9] and sequentially remove species from those at most to least threat of extinction. We find evidence of morphological, but not phylogenetic, homogenization across the avian class, with species becoming more alike in terms of their morphology. We find that most biome and ecoregions are expected to lose morphological diversity at a greater rate than predicted by species loss alone, with the most imperiled regions found in East Asia and the Himalayan uplands and foothills. Only a small proportion of assemblages are threatened with phylogenetic homogenization, in particular parts of Indochina. Species extinctions will lead to a major loss of avian ecological strategies, but not a comparable loss of phylogenetic diversity. As the decline of species with unique traits and their replacement with more widespread generalist species continues, the protection of assemblages at most risk of morphological and phylogenetic homogenization should be a key conservation priority.

RESULTS AND DISCUSSION

Extinction risk across morphospace

Assessing the impact of extinction on both evolutionary and ecological components of biodiversity can reveal the non-random loss of species[10] and highlight where loss of threatened species could lead to biotic homogenization.[7,8,11] This unequal spread of extinction risk across the tree of life[1,12–14] is predicted to lead to an ecological downsizing of species, where the largest, most slow-lived species are lost.[15]

We first examined if bird species at greater risk of extinction have more unique traits. Using a suite of morphological avian traits (beak size and shape, tarsus and wing length, and body size) that are likely to be linked to ecological function and so capture a species ecological niche,[16] we ran a principal components (PCs) analysis and plotted the resultant morphospace based on the first eight PCs (Figure S1; STAR Methods). Avian morphospace is distributed around a dense core of species in the center, with fewer, more diverse forms found towards the edges of morphospace (Figure S1).[9,17,18]

We used data from the International Union for Conservation of Nature (IUCN) Red List[19] to obtain threat statuses for each species and highlight these on the avian morphospace (Figure S1). We calculated the mean distance to centroid of morphospace[20] across morphospace for all bird species, where species from each IUCN threatened category were dropped (critically endangered [CR] > endangered [EN] > vulnerable [VU] > near threatened [NT]) and found a weak trend of species tending to be closer to the center of morphospace (Figure 1). Next, we repeated these calculations on individual PC axes and calculated a standard effect size (SES) for each PC and IUCN threat category (STAR Methods; Table S1). A SES score of < -2 indicates that loss of an IUCN threat category significantly reduces the mean distance to centroid value for that PC.

Generally, as threatened (CR, EN, and VU) species are removed, mean distance to centroid declines significantly more than expected, indicating that threatened species are found at a higher density than non-threatened species at extreme PC values (SES < -2 for majority of PCs; Table S1). Size metrics predominantly load onto PC1 (Table S2), and

 © 2022 The Author(s). Published by Elsevier Inc.
This is an open access article under the CC BY license (http://creativecommons.org/licenses/by/4.0/).

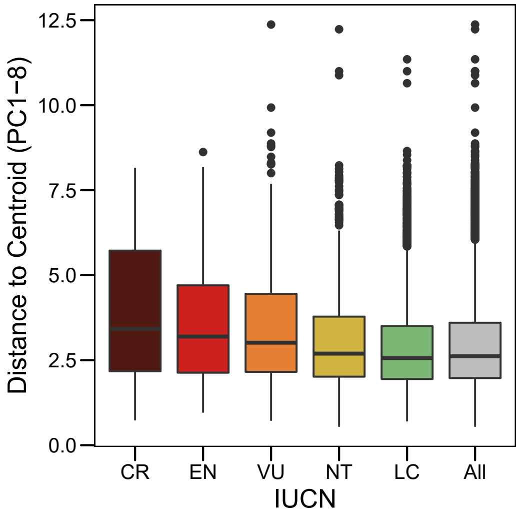

Figure 1. Distance to centroid of morphospace scores for bird species in each IUCN threat category

Distance to centroid scores (the Euclidean distance of each species to the center of morphospace [principal components 1–8]) calculated for all global bird species (All) and for species in each of the IUCN threat categories: critically endangered (CR), endangered (EN), vulnerable (VU), near threatened (NT) and least concern (LC). The higher the mean distance to centroid, the further a species is from the center. 0 is the centroid of morphospace. Box and whiskers show the median value and interquartile range.

See also Figure S1 and Table S1.

our findings support the hypothesis that the largest[10,15,21] and smallest species[14] are likely to be at most risk from extinction (Figure S1; Table S1). Our results suggest that morphological diversity is likely to decrease at a greater rate than expected through species loss alone in the face of global change.[5]

Impacts of extinction on global morphological and phylogenetic diversity

Species at risk of extinction tend to be overrepresented in particular clades and functional groups[22] and belong to evolutionarily unique lineages.[23,24] At a global scale, we predicted that the loss of threatened species will lead to an overall homogenization such that species trait and phylogenetic diversity is lost at a greater rate than expected.

We calculated the mean distance to centroid of morphospace,[20] as a measure of trait diversity and Faith's phylogenetic diversity[3] for all bird species, and where each IUCN category was sequentially dropped (STAR Methods). Morphological and phylogenetic diversity correlate with species richness because the addition of species to a community adds new combinations of traits, as well as a branch length to the community phylogenetic tree.[9,25,26] Therefore, we constructed null models to test whether the species remaining after losing each IUCN category had mean distance to centroid and phylogenetic diversity values that deviated from expected given the observed species richness by calculating SESs (STAR Methods).

We find strong evidence of morphological homogenization across the avian class (SES < -2) (Figure 2). Losing 111 CR species leads to significant homogenization of avian morphospace with a SES score of -7.89 (Figure 2). Morphological homogenization continues with the additional loss of EN (SES = -12.00) and VU (SES = -15.94) species, with no further reduction in SES with the loss of NT (SES = -15.80) species (Figure 2), implying that NT species are lost at random across morphospace, unlike species threatened with extinction (CR, EN, and VU).

We find that the loss of CR, EN, and VU species does not lead to a significant loss of phylogenetic diversity, above that expected through species loss alone (SES > -2: Figure 2). Only the additional loss of NT species results in a significant reduction in phylogenetic diversity (SES = -3.39: Figure 2), indicating that NT species are more evolutionarily distinct compared to the global pool of species. Our findings of a lack of congruence between morphological and phylogenetic diversity loss across the avian class indicates that species threatened with extinction exhibit traits that are more unique, given their phylogenetic history, compared to the wider species pool.

Both trait and phylogenetic diversity measures are amassed over long evolutionary timespans and are often considered to be positively correlated.[27] This occurs where trait evolution is phylogenetically constrained such that species traits exhibit strong phylogenetic signal and diverge over time (e.g., following Brownian motion).[27,28] Therefore, the extinction of an evolutionarily old species with no close relatives that has evolved unique traits could have a greater impact on phylogenetic and trait diversity than a more recently evolved species with many close relatives with similar trait values.[5,29] However, not all species traits evolve at a constant rate (e.g. in the work of Chira and Thomas, Harmon et al., O'Meara et al., and Venditti et al.[30–33]) or show strong phylogenetic signal,[34] and this could therefore lead to the differences in morphological and phylogenetic diversity loss that we find across the avian class.

To assess the relationship between morphological diversity and phylogenetic history, we tested for multivariate phylogenetic signal across our morphological traits. We find a strong multivariate phylogenetic signal across our eight PCs. However, we find significant departure from strict Brownian motion with a mean $\lambda = 0.920$ (lower confidence interval = 0.918, upper confidence interval = 0.923) across 200 out of 200 phylogenetic trees. Moreover, previous studies on subsets of the data show widespread variation in the rate of evolution.[17,35] Together, this indicates that morphological and phylogenetic diversity are at least partially decoupled and that phylogenetic diversity loss is not always an appropriate surrogate for morphological diversity loss.[5,36,37]

Spatial loss of morphological and phylogenetic diversity

Patterns of trait and phylogenetic homogenization are also likely to vary across space. Raw phylogenetic and trait diversity are distributed unequally globally,[9,25,26,38,39] while threats faced (e.g., habitat loss, hunting, or climate change) and species sensitivities to these threats are spatially variable and increasing in intensity due to human activities.[6] For example, the greatest threats to tropical terrestrial vertebrates are logging and agriculture, whereas the threats posed by invasive species are particularly high for island birds.[40,41] Thus, certain regions will be at increased risk from trait and phylogenetic homogenization.[6] To examine this, we focus on bird communities found in each of the world's ecoregions (n = 814)—units of land that contain distinct assemblages of natural communities, species,

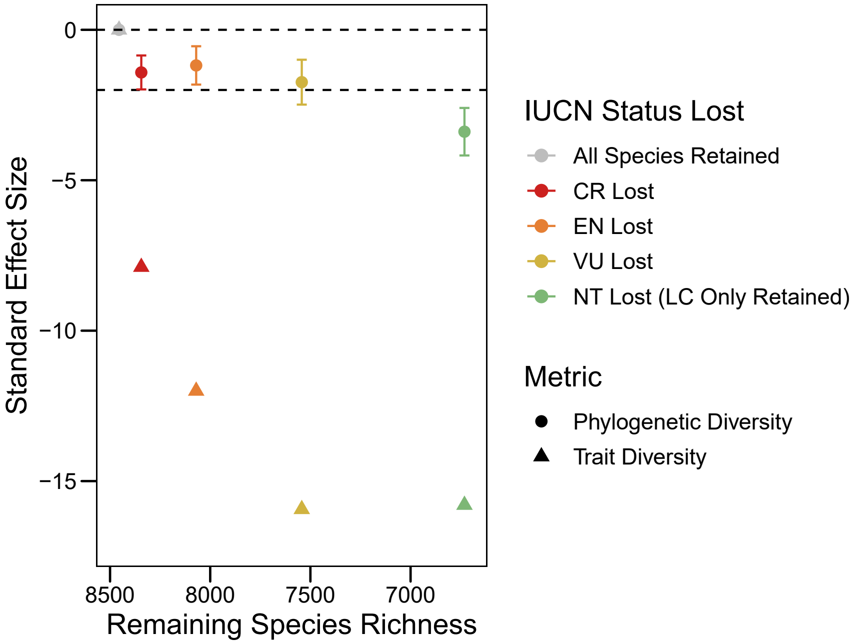

Figure 2. Variation in mean distance to centroid and phylogenetic diversity where each IUCN category is lost across the entire avian class

The standard effect size of phylogenetic diversity (circles) and trait diversity (mean distance to centroid) (triangles) calculated for the whole global species pool of birds (grey, n = 8,455) and for each remaining value of species richness where species categorized under each IUCN threat status are lost: critically endangered (CR: red) species, then endangered (EN: orange) species, vulnerable (VU: yellow) species, and finally near threatened (NT: green) species, leaving least concern (LC) species only. Error bars show the standard deviation of phylogenetic diversity calculated on 200 phylogenetic trees. The dotted lines are where SES = 0 and SES = −2. Values < −2 indicate significant homogenization.

dynamics, and environmental conditions—and biomes (n = 14)—major habitat types (e.g., tropical grassland).[42] We calculate the mean distance to centroid, phylogenetic diversity, and the SES of both metrics for each biome and ecoregion[42] communities[43] after losing CR, EN, VU, and finally NT bird species (STAR Methods).

We find strong latitudinal variation in biome morphological diversity and phylogenetic diversity, with assemblages in the tropics harboring the highest phylogenetic diversity and being particularly clustered around the centroid of morphospace (Figures 3A and 3B). If CR species went extinct, 12 of the 14 biomes (86%) would experience morphological homogenization (SES < −2), with the most imperiled biomes being tropical dry and moist forests and flooded grasslands (Figure 3C). All biomes would experience homogenization with the further loss of EN, VU, and NT species (Figures 3E and S2), with the montane grassland biome becoming especially highly threatened with the loss of EN species (Figure 3E).

Phylogenetic diversity loss does not show significant homogenization for most biomes when CR species are lost (13 out of 14), with only Mediterranean forests experiencing exceptional homogenization (Figure 3D). Likewise, when EN species are additionally lost, only the temperate broadleaf forest biome is threatened with phylogenetic homogenization (Figure 3F). For both biomes, homogenization is only just significant.

We further find low morphological diversity in many East Asian ecoregions. The highest morphological diversity is found across ecoregions in New Zealand and the southern tip of South America, as well as northern North America (Figure 4A). Many ecoregions of the world would experience morphological homogenization (mean distance to centroid SES < −2) if species in each IUCN category were to go extinct (Figures 4 and S2). For example, 48.4% of ecoregions would experience morphological homogenization where CR species are lost (n = 382 ecoregions; Figure 4C). Ecoregions that are particularly morphologically imperiled are those found in the Himalayas and parts of Indochina (Figures 4C and 4E), with the addition of ecoregions across sub-Saharan and East Africa where VU and NT species morphology is lost (Figure S2E). Many island systems (e.g., Hawaii, French Polynesia, and Madagascar) would experience significant morphological homogenization when losing the most threatened species (Figure S4C). Island taxa are amongst the most threatened globally, and significant losses of iconic, morphologically diverse species have already occurred (e.g., many Hawaiian honeycreepers or the elephant bird), resulting in homogenization of trait diversity.[44]

Fewer ecoregions would experience phylogenetic homogenization (SES < −2) where CR (5.5% ecoregions, n = 382) and CR and EN species (4.3% ecoregions, n = 698) are lost (Figures 4D and 4F). The most phylogenetically imperiled ecoregions are found in parts of Indochina, particularly Cambodia and Vietnam, as well as French Polynesia, Iberian and Pyrenean montane forests, and Australia (Figures 4D and 4F). Further loss of VU and NT species would lead to the addition of central African ecoregions being threatened with phylogenetic homogenization, as well as those regions covering the length of the Andes and Sulawesi (Figures S2F and S2H).

Our finding that morphological, but not phylogenetic, homogenization is an inevitable outcome of predicted biodiversity loss for the majority of biomes and ecoregions highlights the potential for ecological changes that could lead to a considerable loss of ecological roles and ecosystem functioning, productivity, and services.[7] Of six CR species lost in the top five most imperiled ecoregions, four are vultures (*Sarcogyps calvus*, *Gyps tenuirostris*, *bengalensis*, *and indicus*). The traits used in this study are broadly similar to those linked to the ecological foraging guilds of birds,[16,18] and vultures, as large-bodied, obligate scavengers, fill distinct areas of morphospace.[9,45] Therefore, it is likely that the considerable loss of morphological diversity in the Himalayan ecoregions is partly driven by the loss of vultures—the most imperiled group of birds.[46] Vultures provide vital ecosystem services by removing decaying carcasses, which could otherwise increase the direct transmission of infectious diseases[47–49] and

CellPress
OPEN ACCESS

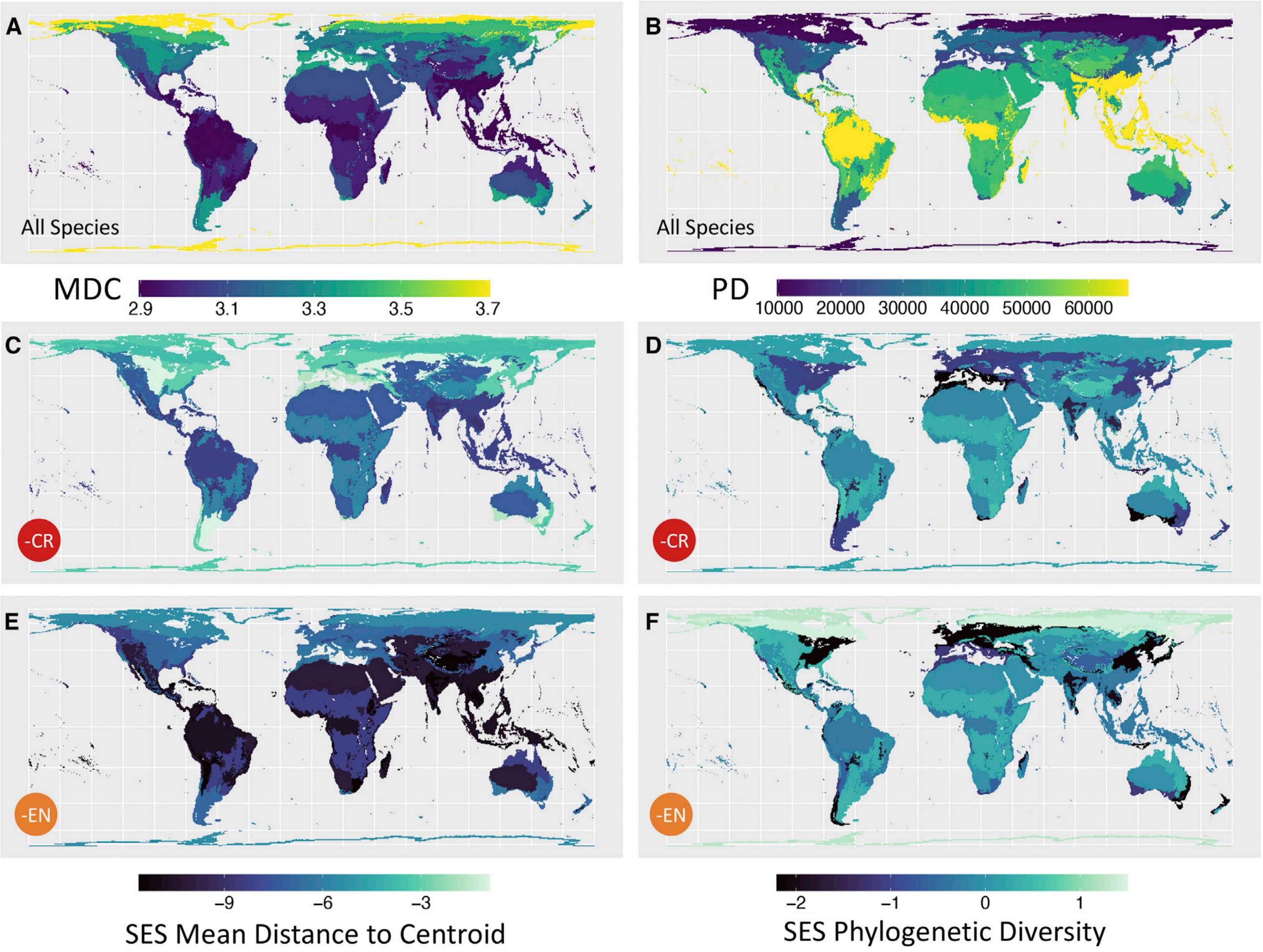

Figure 3. Variation in morphological diversity and phylogenetic diversity across avian assemblages in each terrestrial biome

(A and B) The amount of raw morphological (mean distance to centroid) (A) and phylogenetic diversity (B) for 8,426 bird species across 14 terrestrial biomes. The darker blue color indicates that species in that biome are on average closer to the center of morphospace (A) and have low phylogenetic diversity. The lighter yellow color indicates that species in that biome tend to be further from the center of morphospace (A) and have high phylogenetic diversity.

(C–F) Standard effect sizes (SES) for morphological (C) and phylogenetic diversity (D) were calculated from 1,000 simulated biome communities after critically endangered (CR) species and, additionally, when endangered (EN) species were dropped (E and F). The darkest blue color indicates where SES values are more negative than expected, with values < -2 showing significant homogenization.

See also Figures S2 and S3.

increase populations of opportunistic scavengers (i.e., dogs and rats) that spread rabies and bubonic plague.[47,50]

Another region containing assemblages at risk of morphological homogenization are the dry and moist forest ecoregions of South Vietnam and Cambodia, where there is also exceptionally high expected loss of phylogenetic diversity. The CR and EN species present are therefore likely to be phylogenetically unique and exhibit sets of traits that the surviving species pool does not contain. Indeed, highly threatened species here are amongst the highest evolutionarily distinct and globally endangered[51] (EDGE[52]) classified species including giant ibis (*Thaumatibis gigantea*, ranked second by EDGE), Bengal florican (*Houbaropsis bengalensis*, seventh), and white-shouldered ibis (*Pseudibis davisoni*, sixteenth). Despite phylogenetic diversity increasingly being stated as an essential facet of biodiversity to conserve to meet global targets of biodiversity conservation (e.g., the 2019 report from IPBES[53]), these species are currently only receiving low, medium, and very low conservation attention, respectively.[52]

Despite being less widespread than morphological diversity loss, phylogenetic diversity loss remains an important metric for assessing the impact of species extinction.[3] Specific sets of traits are used to capture morphological diversity that are expected to relate to specific ecological niches and functions in the present day,[54] but it is impossible to capture all possible combinations of traits that species represent to exactly map form to function.[3] Phylogenetic diversity captures this feature diversity, including traits not currently known or measurable.[3,55] In turn, this makes phylogenetic diversity a good indicator of biodiversity "option value"—the unknown future benefits to humans not currently realized.[3] Using subsets of ecologically relevant traits captures the impacts of species loss on specific aspects of phenotype, which may be important to conserve if they link to

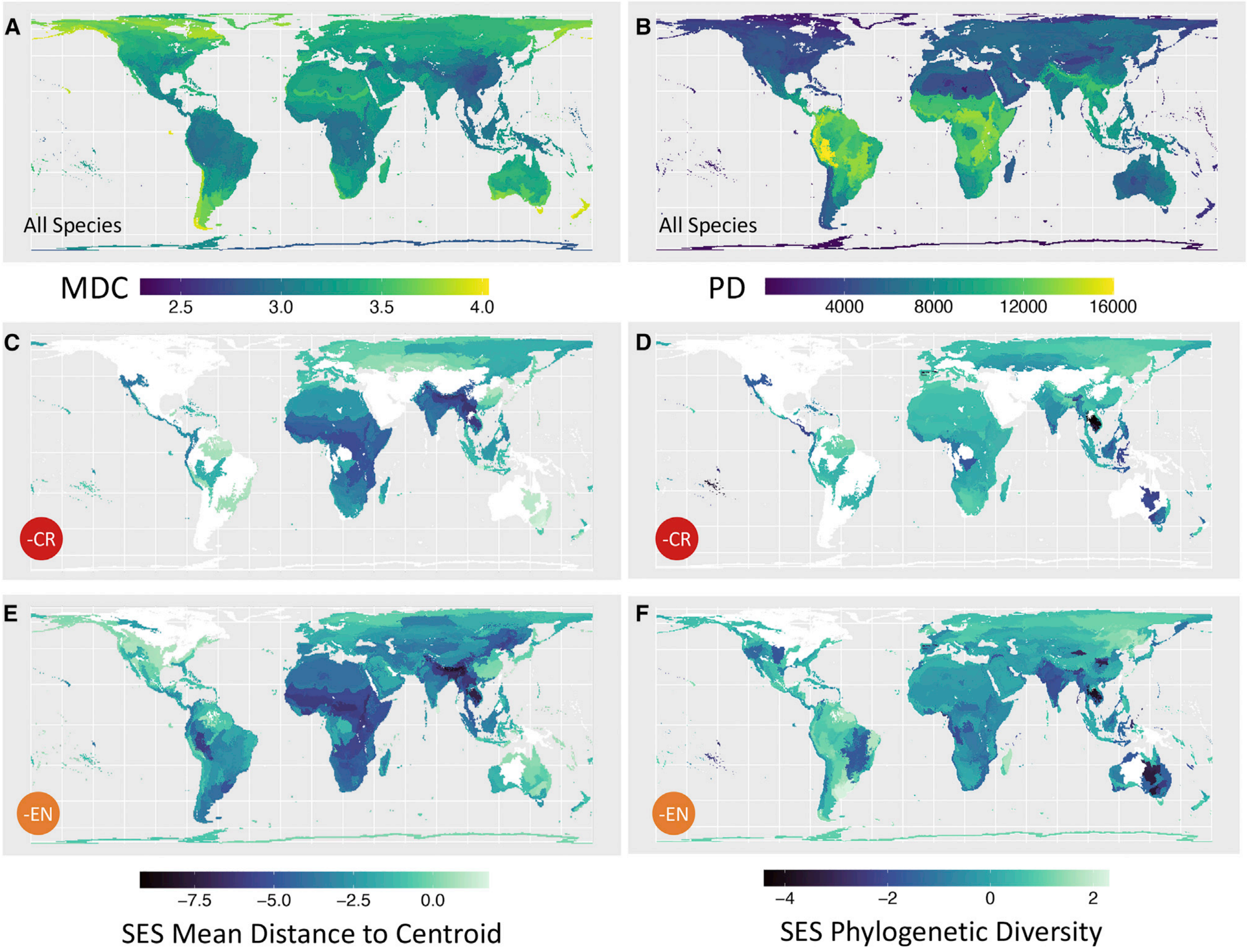

Figure 4. Variation in morphological diversity and phylogenetic diversity across avian assemblages in each terrestrial ecoregion

(A and B) The amount of raw morphological (mean distance to centroid) (A) and phylogenetic diversity (B) for 8,423 bird species across 814 terrestrial ecoregions. The darker blue color indicates that species in that ecoregion are on average closer to the center of morphospace (A) and have low phylogenetic diversity. The lighter yellow color indicates that species in that ecoregion tend to be further from the center of morphospace (A) and have high phylogenetic diversity.

(C–F) Standard effect sizes (SES) for morphological (C) and phylogenetic diversity (D) were calculated from 1,000 simulated biome communities after critically endangered (CR) species and, additionally, when endangered (EN) species were dropped (E and F). The darkest blue color indicates where SES values are more negative than expected, with values < -2 showing significant homogenization. White ecoregions are those where no CR or EN species are present, and therefore, SES scores cannot be calculated.

See also Figures S2 and S4.

key aspects of ecosystem functioning or services.[56] Priority should therefore be given to establishing whether measurable species traits can more directly capture important features to conserve than phylogeny.

Our study focuses on species extinctions as a primary driver of morphological and phylogenetic homogenization.[11] While we capture the range expansion of species to present day, including reintroduced species ranges, we do not include species introduced through direct or indirect human activity. The introduction and spread of non-native species are another key driver of the biological extinction crisis,[57] as they tend to more generalist[7,11] and can diminish the distinctiveness of regional assemblages, reducing trait and phylogenetic differences between species.[8,44,58,59] Furthermore, we deal with global extinction, but not local extirpation. In many areas, species are already functionally extinct across most of their ranges, and so morphological diversity is already likely to be dramatically constrained.[60] Given that the replacement of more specialist species by a smaller number of more generalist species[7,11] is unlikely to abate, as well as increasing pressure from additional drivers of species decline and distribution change (e.g., climate change,[1] wildlife trade,[61] etc.), it is likely that our findings underestimate the degree of morphological homogenization that will and has already occurred during the Anthropocene.

In conclusion, our work reveals widespread morphological homogenization across the entire avian class, most terrestrial biomes, and half of all ecoregions. The predicted loss of morphological diversity exceeds that expected if future extinctions were random and highlights important losses of ecological function

across assemblages, with important ramifications for humans as ecosystem services are lost. Phylogenetic diversity tends to decline as expected as species go extinct. Whether measurable species traits can capture features of conservation priority, such as key ecosystem services, more directly is crucial to understand when assessing the impacts of extinction on biodiversity.

STAR★METHODS

Detailed methods are provided in the online version of this paper and include the following:

- KEY RESOURCES TABLE
- RESOURCE AVAILABILITY
  - Lead contact
  - Materials availability
  - Data and code availability
- EXPERIMENTAL MODEL AND SUBJECT DETAILS
  - Morphological trait space
  - Threat status
  - Species pools
- METHOD DETAILS
  - Avian morphological and phylogenetic diversity measures
- QUANTIFICATION AND STATISTICAL ANALYSIS
  - Phylogenetic signal across morphological traits
  - Simulating the impact of threatened species loss on morphological and phylogenetic diversity

SUPPLEMENTAL INFORMATION

Supplemental information can be found online at https://doi.org/10.1016/j.cub.2022.06.018.

ACKNOWLEDGMENTS

We thank J. Bright, E. Capp, C. Cooney, and Z. Varley, who, alongside E.C.H. and G.H.T., collected data from museum specimens and designed analytical protocols for producing the beak shape dataset. We thank M. Adams, H. van Grouw, and R. Prys-Jones from Bird Group at the Natural History Museum, Tring; H. McGhie at the Manchester Museum; and S. Hackett and B. Marks at the Field Museum, Chicago for providing access to and expertise in the ornithological collections and the volunteer citizen scientists who landmarked 3D beak scans at http://www.markmybird.org. E.C.H. was funded by a NERC PhD studentship under the Adapting to the Challenges of a Changing Environment (ACCE) DTP (NE/L002450/1). G.H.T was funded by the European Research Council (grant number 615709 project "ToLERates") and a Royal Society University Research Fellowship (UF120016 and URF\R\180006).

AUTHOR CONTRIBUTIONS

E.C.H., D.P.E., and G.H.T. conceived the ideas and designed methodology; E.C.H. and G.H.T., collected data from museum specimens and designed analytical protocols for producing the beak shape dataset; E.C.H. analyzed the data and led the writing of the manuscript. All authors contributed critically to the drafts and gave final approval for publication.

DECLARATION OF INTERESTS

The authors declare no competing interests.

INCLUSION AND DIVERSITY

One or more of the authors of this paper self-identifies as living with a disability. While citing references scientifically relevant for this work, we also actively worked to promote gender balance in our reference list.

Received: March 16, 2022
Revised: May 11, 2022
Accepted: June 8, 2022
Published: July 21, 2022

REFERENCES

1. Dirzo, R., Young, H.S., Galetti, M., Ceballos, G., Isaac, N.J.B., and Collen, B. (2014). Defaunation in the Anthropocene. Science *345*, 401–406. https://doi.org/10.1126/science.1251817.
2. Devictor, V., Mouillot, D., Meynard, C., Jiguet, F., Thuiller, W., and Mouquet, N. (2010). Spatial mismatch and congruence between taxonomic, phylogenetic and functional diversity: the need for integrative conservation strategies in a changing world. Ecol. Lett. *13*, 1030–1040. https://doi.org/10.1111/j.1461-0248.2010.01493.x.
3. Faith, D.P. (1992). Conservation evaluation and phylogenetic diversity. Biol. Conserv. *61*, 1–10. https://doi.org/10.1016/0006-3207(92)91201-3.
4. Purvis, A., and Hector, A. (2000). Getting the measure of biodiversity. Nature *405*, 212–219. https://doi.org/10.1038/35012221.
5. Oliveira, B.F., Scheffers, B.R., and Costa, G.C. (2020). Decoupled erosion of amphibians' phylogenetic and functional diversity due to extinction. Global Ecol. Biogeogr. *29*, 309–319. https://doi.org/10.1111/geb.13031.
6. Brodie, J.F., Williams, S., and Garner, B. (2021). The decline of mammal functional and evolutionary diversity worldwide. Proc. Natl. Acad. Sci. USA *118*. e1921849118. https://doi.org/10.1073/pnas.1921849118.
7. Clavel, J., Julliard, R., and Devictor, V. (2011). Worldwide decline of specialist species: toward a global functional homogenization? Front. Ecol. Environ. *9*, 222–228. https://doi.org/10.1890/080216.
8. Daru, B.H., Davies, T.J., Willis, C.G., Meineke, E.K., Ronk, A., Zobel, M., Pärtel, M., Antonelli, A., and Davis, C.C. (2021). Widespread homogenization of plant communities in the Anthropocene. Nat. Commun. *12*, 6983. https://doi.org/10.1038/s41467-021-27186-8.
9. Hughes, E.C., Edwards, D.P., Bright, J.A., Capp, E.J.R., Cooney, C.R., Varley, Z.K., and Thomas, G.H. (2022). Global biogeographic patterns of avian morphological diversity. Ecol. Lett. *25*, 598–610. https://doi.org/10.1111/ele.13905.
10. Cardillo, M., Mace, G.M., Jones, K.E., Bielby, J., Bininda-Emonds, O.R.P., Sechrest, W., Orme, C.D.L., and Purvis, A. (2005). Multiple Causes of High Extinction Risk in Large Mammal Species. Science *309*, 1239–1241. https://doi.org/10.1126/science.1116030.
11. McKinney, M.L., and Lockwood, J.L. (1999). Biotic homogenization: a few winners replacing many losers in the next mass extinction. Trends Ecol. Evol. *14*, 450–453. https://doi.org/10.1016/S0169-5347(99)01679-1.
12. Cardillo, M., Mace, G.M., Gittleman, J.L., Jones, K.E., Bielby, J., and Purvis, A. (2008). The predictability of extinction: biological and external correlates of decline in mammals. Proc. Biol. Sci. *275*, 1441–1448. https://doi.org/10.1098/rspb.2008.0179.
13. Lee, T.M., and Jetz, W. (2011). Unravelling the structure of species extinction risk for predictive conservation science. Proc. Biol. Sci. *278*, 1329–1338. https://doi.org/10.1098/rspb.2010.1877.
14. Ripple, W.J., Wolf, C., Newsome, T.M., Hoffmann, M., Wirsing, A.J., and McCauley, D.J. (2017). Extinction risk is most acute for the world's largest and smallest vertebrates. Proc. Natl. Acad. Sci. USA *114*, 10678–10683. https://doi.org/10.1073/pnas.1702078114.
15. Cooke, R.S.C., Eigenbrod, F., and Bates, A.E. (2019). Projected losses of global mammal and bird ecological strategies. Nat. Commun. *10*, 2279. https://doi.org/10.1038/s41467-019-10284-z.
16. Pigot, A.L., Trisos, C.H., and Tobias, J.A. (2016). Functional traits reveal the expansion and packing of ecological niche space underlying an

elevational diversity gradient in passerine birds. Proc. Biol. Sci. *283*, 20152013. https://doi.org/10.1098/rspb.2015.2013.

17. Chira, A.M., Cooney, C.R., Bright, J.A., Capp, E.J.R., Hughes, E.C., Moody, C.J.A., Nouri, L.O., Varley, Z.K., and Thomas, G.H. (2018). Correlates of rate heterogeneity in avian ecomorphological traits. Ecol. Lett. *21*, 1505–1514. https://doi.org/10.1111/ele.13131.
18. Pigot, A.L., Sheard, C., Miller, E.T., Bregman, T.P., Freeman, B.G., Roll, U., Seddon, N., Trisos, C.H., Weeks, B.C., and Tobias, J.A. (2020). Macroevolutionary convergence connects morphological form to ecological function in birds. Nature Ecology Evolution *4*, 230–239. https://doi.org/10.1038/s41559-019-1070-4.
19. IUCN (2020). The IUCN Red List of threatened species. http://www.iucnredlist.org.
20. Laliberté, E., and Legendre, P. (2010). A distance-based framework for measuring functional diversity from multiple traits. Ecology *91*, 299–305. https://doi.org/10.1890/08-2244.1.
21. Gaston, K.J., and Blackburn, T.M. (1995). Birds, body size and the threat of extinction. Phil. Trans. R. Soc. Lond. B *347*, 205–212. https://doi.org/10.1098/rstb.1995.0022.
22. Purvis, A., Gittleman, J.L., Cowlishaw, G., and Mace, G.M. (2000). Predicting extinction risk in declining species. Proc. Biol. Sci. *267*, 1947–1952. https://doi.org/10.1098/rspb.2000.1234.
23. Murali, G., Gumbs, R., Meiri, S., and Roll, U. (2021). Global determinants and conservation of evolutionary and geographic rarity in land vertebrates. Sci. Adv. *7*, eabe5582. https://doi.org/10.1126/sciadv.abe5582.
24. Vamosi, J.C., and Wilson, J.R.U. (2008). Nonrandom extinction leads to elevated loss of angiosperm evolutionary history. Ecol. Lett. *11*, 1047–1053. https://doi.org/10.1111/j.1461-0248.2008.01215.x.
25. Safi, K., Cianciaruso, M.V., Loyola, R.D., Brito, D., Armour-Marshall, K., and Diniz-Filho, J.A.F. (2011). Understanding global patterns of mammalian functional and phylogenetic diversity. Phil. Trans. Biol. Sci. *366*, 2536–2544. https://doi.org/10.1098/rstb.2011.0024.
26. Voskamp, A., Baker, D.J., Stephens, P.A., Valdes, P.J., and Willis, S.G. (2017). Global patterns in the divergence between phylogenetic diversity and species richness in terrestrial birds. J. Biogeogr. *44*, 709–721. https://doi.org/10.1111/jbi.12916.
27. Wiens, J.J., and Graham, C.H. (2005). Niche Conservatism: Integrating Evolution, Ecology, and Conservation Biology. Annu. Rev. Ecol. Evol. Syst. *36*, 519–539. https://doi.org/10.1146/annurev.ecolsys.36.102803.095431.
28. Felsenstein, J. (1985). Phylogenies and the Comparative Method. Am. Nat. *125*, 1–15. https://doi.org/10.1086/284325.
29. Redding, D.W., DeWOLFF, C.V., and Mooers, A.Ø. (2010). Evolutionary Distinctiveness, Threat Status, and Ecological Oddity in Primates. Conserv. Biol. *24*, 1052–1058. https://doi.org/10.1111/j.1523-1739.2010.01532.x.
30. Chira, A.M., and Thomas, G.H. (2016). The impact of rate heterogeneity on inference of phylogenetic models of trait evolution. J. Evol. Biol. *29*, 2502–2518. https://doi.org/10.1111/jeb.12979.
31. Harmon, L.J., Losos, J.B., Jonathan Davies, T., Gillespie, R.G., Gittleman, J.L., Bryan Jennings, W., Kozak, K.H., McPeek, M.A., Moreno-Roark, F., Near, T.J., et al. (2010). Early Bursts of Body Size and Shape Evolution Are Rare in Comparative Data. Evolution *64*, 2385–2396. https://doi.org/10.1111/j.1558-5646.2010.01025.x.
32. O'Meara, B.C., Ané, C., Sanderson, M.J., and Wainwright, P.C. (2006). Testing for Different Rates of Continuous Trait Evolution Using Likelihood. Evolution *60*, 922–933. https://doi.org/10.1111/j.0014-3820.2006.tb01171.x.
33. Venditti, C., Meade, A., and Pagel, M. (2011). Multiple routes to mammalian diversity. Nature *479*, 393–396. https://doi.org/10.1038/nature10516.
34. Losos, J.B. (2008). Phylogenetic niche conservatism, phylogenetic signal and the relationship between phylogenetic relatedness and ecological similarity among species. Ecol. Lett. *11*, 995–1003. https://doi.org/10.1111/j.1461-0248.2008.01229.x.
35. Cooney, C.R., Bright, J.A., Capp, E.J.R., Chira, A.M., Hughes, E.C., Moody, C.J.A., Nouri, L.O., Varley, Z.K., and Thomas, G.H. (2017). Mega-evolutionary dynamics of the adaptive radiation of birds. Nature *542*, 344–347. https://doi.org/10.1038/nature21074.
36. Kelly, S., Grenyer, R., and Scotland, R.W. (2014). Phylogenetic trees do not reliably predict feature diversity. Divers. Distrib. *20*, 600–612. https://doi.org/10.1111/ddi.12188.
37. Mazel, F., Pennell, M.W., Cadotte, M.W., Diaz, S., Dalla Riva, G.V., Grenyer, R., Leprieur, F., Mooers, A.O., Mouillot, D., Tucker, C.M., and Pearse, W.D. (2018). Prioritizing phylogenetic diversity captures functional diversity unreliably. Nat. Commun. *9*, 2888. https://doi.org/10.1038/s41467-018-05126-3.
38. McLean, M., Stuart-Smith, R.D., Villéger, S., Auber, A., Edgar, G.J., MacNeil, M.A., Loiseau, N., Leprieur, F., and Mouillot, D. (2021). Trait similarity in reef fish faunas across the world's oceans. Proc. Natl. Acad. Sci. USA *118*. e2012318118. https://doi.org/10.1073/pnas.2012318118.
39. Sheard, C., Neate-Clegg, M.H.C., Alioravainen, N., Jones, S.E.I., Vincent, C., MacGregor, H.E.A., Bregman, T.P., Claramunt, S., and Tobias, J.A. (2020). Ecological drivers of global gradients in avian dispersal inferred from wing morphology. Nat. Commun. *11*, 2463. https://doi.org/10.1038/s41467-020-16313-6.
40. Davies, T.J. (2019). The macroecology and macroevolution of plant species at risk. New Phytol. *222*, 708–713. https://doi.org/10.1111/nph.15612.
41. Harfoot, M.B.J., Johnston, A., Balmford, A., Burgess, N.D., Butchart, S.H.M., Dias, M.P., Hazin, C., Hilton-Taylor, C., Hoffmann, M., Isaac, N.J.B., et al. (2021). Using the IUCN Red List to map threats to terrestrial vertebrates at global scale. Nat. Ecol. Evol. *5*, 1510–1519. https://doi.org/10.1038/s41559-021-01542-9.
42. Olson, D.M., Dinerstein, E., Wikramanayake, E.D., Burgess, N.D., Powell, G.V.N., Underwood, E.C., D'amico, J.A., Itoua, I., Strand, H.E., Morrison, J.C., et al. (2001). Terrestrial Ecoregions of the World: A New Map of Life on Earth: A new global map of terrestrial ecoregions provides an innovative tool for conserving biodiversity. Bioscience *51*, 933–938. https://doi.org/10.1641/0006-3568(2001)051[0933:TEOTWA]2.0.CO;2.
43. Birdlife International. (2020). Data Zone. http://www.datazone.birdlife.org.
44. Sayol, F., Cooke, R.S.C., Pigot, A.L., Blackburn, T.M., Tobias, J.A., Steinbauer, M.J., Antonelli, A., and Faurby, S. (2021). Loss of functional diversity through anthropogenic extinctions of island birds is not offset by biotic invasions. Sci. Adv. *7*, eabj5790. https://doi.org/10.1126/sciadv.abj5790.
45. Bright, J.A., Marugán-Lobón, J., Cobb, S.N., and Rayfield, E.J. (2016). The shapes of bird beaks are highly controlled by nondietary factors. Proc. Natl. Acad. Sci. USA *113*, 5352–5357. https://doi.org/10.1073/pnas.1602683113.
46. Buechley, E.R., and Şekercioğlu, Ç.H. (2016). The avian scavenger crisis: Looming extinctions, trophic cascades, and loss of critical ecosystem functions. Biol. Conserv. *198*, 220–228. https://doi.org/10.1016/j.biocon.2016.04.001.
47. DeVault, T.L., Beasley, J.C., Olson, Z.H., Moleón, M., Carrete, M., Margalida, A., and Sánchez-Zapata, J.A. (2016). Ecosystem Services Provided by Avian Scavengers. In Why Birds Matter: Avian Ecological Function and Ecosystem Services, Ç. Sekercioglu, D.G. Wenny, and C.J. Whelan, eds. (University of Chicago Press), pp. 235–270. https://doi.org/10.7208/9780226382777.
48. Moleón, M., Sánchez-Zapata, J.A., Margalida, A., Carrete, M., Owen-Smith, N., and Donázar, J.A. (2014). Humans and Scavengers: The Evolution of Interactions and Ecosystem Services. Bioscience *64*, 394–403. https://doi.org/10.1093/biosci/biu034.
49. Ogada, D.L., Torchin, M.E., Kinnaird, M.F., and Ezenwa, V.O. (2012). Effects of vulture declines on facultative scavengers and potential implications for mammalian disease transmission. Conserv. Biol. *26*, 453–460. https://doi.org/10.1111/j.1523-1739.2012.01827.x.
50. Markandya, A., Taylor, T., Longo, A., Murty, M.N., Murty, S., and Dhavala, K. (2008). Counting the cost of vulture decline—An appraisal of the human

CellPress
OPEN ACCESS

health and other benefits of vultures in India. Ecol. Econ. *67*, 194–204. https://doi.org/10.1016/j.ecolecon.2008.04.020.

51. Jetz, W., Thomas, G.H., Joy, J.B., Redding, D.W., Hartmann, K., and Mooers, A.O. (2014). Global distribution and conservation of evolutionary distinctness in birds. Curr. Biol. *24*, 919–930. https://doi.org/10.1016/j.cub.2014.03.011.
52. EDGE of Existence (2022). Top 100 EDGE Birds. http://www.edgeofexistence.org/birds/.
53. IPBES (2019). In Global assessment report on biodiversity and ecosystem services of the Intergovernmental Science-Policy Platform on Biodiversity and Ecosystem Services, E.S. Brondizio, J. Settele, S. Díaz, and H.T. Ngo, eds. (IPBES Secretariat), p. 1148. https://doi.org/10.5281/zenodo.3831673.
54. Petchey, O.L., and Gaston, K.J. (2006). Functional diversity: back to basics and looking forward. Ecol. Lett. *9*, 741–758. https://doi.org/10.1111/j.1461-0248.2006.00924.x.
55. Faith, D.P. (2008). Threatened Species and the Potential Loss of Phylogenetic Diversity: Conservation Scenarios Based on Estimated Extinction Probabilities and Phylogenetic Risk Analysis. Conserv. Biol. *22*, 1461–1470. https://doi.org/10.1111/j.1523-1739.2008.01068.x.
56. Flynn, D.F.B., Mirotchnick, N., Jain, M., Palmer, M.I., and Naeem, S. (2011). Functional and phylogenetic diversity as predictors of biodiversity–ecosystem-function relationships. Ecology *92*, 1573–1581. https://doi.org/10.1890/10-1245.1.
57. Blackburn, T.M., Bellard, C., and Ricciardi, A. (2019). Alien versus native species as drivers of recent extinctions. Front. Ecol. Environ. *17*, 203–207. https://doi.org/10.1002/fee.2020.
58. Socolar, J.B., Gilroy, J.J., Kunin, W.E., and Edwards, D.P. (2016). How should beta-diversity inform biodiversity conservation? Trends Ecol. Evol. *31*, 67–80. https://doi.org/10.1016/j.tree.2015.11.005.
59. Yang, Q., Weigelt, P., Fristoe, T.S., Zhang, Z., Kreft, H., Stein, A., Seebens, H., Dawson, W., Essl, F., König, C., et al. (2021). The global loss of floristic uniqueness. Nat. Commun. *12*, 7290. https://doi.org/10.1038/s41467-021-27603-y.
60. Anderson, S.H., Kelly, D., Ladley, J.J., Molloy, S., and Terry, J. (2011). Cascading Effects of Bird Functional Extinction Reduce Pollination and Plant Density. Science *331*, 1068–1071. https://doi.org/10.1126/science.1199092.
61. Morton, O., Scheffers, B.R., Haugaasen, T., and Edwards, D.P. (2021). Impacts of wildlife trade on terrestrial biodiversity. Nat Ecol Evol *5*, 540–548. https://doi.org/10.1038/s41559-021-01399-y.
62. Hughes, E.C., Edwards, D.P., and Thomas, G.H. (2022). Data from: The homogenisation of avian morphological and phylogenetic diversity under the global extinction crisis (The University of Sheffield). Dataset. ORDA provided by Figshare. https://doi.org/10.15131/shef.data.20004806.v1.
63. Core Team, R. (2021). R: A language and environment for statistical computing (R Foundation for Statistical Computing).
64. RStudio Team (2021). RStudio: Integrated Development for R (RStudio, PBC).
65. Jetz, W., Thomas, G.H., Joy, J.B., Hartmann, K., and Mooers, A.O. (2012). The global diversity of birds in space and time. Nature *491*, 444–448. https://doi.org/10.1038/nature11631.
66. Wilman, H., Belmaker, J., Simpson, J., de la Rosa, C., Rivadeneira, M.M., and Jetz, W. (2014). EltonTraits 1.0: Species-level foraging attributes of the world's birds and mammals. Ecology *95*, 2027. https://doi.org/10.1890/13-1917.1.
67. Guillerme, T. (2018). dispRity: A modular R package for measuring disparity. Methods Ecol. Evol. *9*, 1755–1763. https://doi.org/10.1111/2041-210X.13022.
68. Tsirogiannis, C., and Sandel, B. (2016). PhyloMeasures: a package for computing phylogenetic biodiversity measures and their statistical moments. Ecography *39*, 709–714. https://doi.org/10.1111/ecog.01814.
69. Hackett, S.J., Kimball, R.T., Reddy, S., Bowie, R.C.K., Braun, E.L., Braun, M.J., Chojnowski, J.L., Cox, W.A., Han, K.L., Harshman, J., Huddleston, C.J., Marks, B.D., Miglia, K.J., Moore, W.S., Sheldon, F.H., Steadman, D.W., Witt, C.C., Yuri, T., and Harshman, J. (2008). A phylogenomic study of birds reveals their evolutionary history. Science *320*, 1763–1768. https://doi.org/10.1126/science.1157704.
70. Puttick, M.N., Ingram, T., Clarke, M., and Thomas, G.H. (2020). MOTMOT: Models of trait macroevolution on trees (an update). Methods Ecol. Evol. *11*, 464–471. https://doi.org/10.1111/2041-210X.13343.
71. Pagel, M. (1997). Inferring evolutionary processes from phylogenies. Zool. Scripta *26*, 331–348. https://doi.org/10.1111/j.1463-6409.1997.tb00423.x.
72. Pagel, M. (1999). Inferring the historical patterns of biological evolution. Nature *401*, 877–884. https://doi.org/10.1038/44766.

STAR★METHODS

KEY RESOURCES TABLE

REAGENT or RESOURCE	SOURCE	IDENTIFIER
Deposited data		
Original data and code	Hughes et al.[62]	https://doi.org/10.15131/shef.data.20004806.v1
Software and algorithms		
R Version 4.1.1	The R Foundation for Statistical Computing[63]	https://cran.r-project.org
R Studio Version 1.4.1717	RStudio[64]	https://rstudio.com/products/rstudio/download/
Other		
Global bird species distribution maps	Birdlife International[43]	http://datazone.birdlife.org/home
Avian taxonomy	Wilman et al.[65]	http://birdtree.org/
IUCN Red List categories	IUCN[19]	https://www.iucnredlist.org/
Bird traits	Hughes et al.[9] and Wilman et al.[66]	https://doi.org/10.15131/ shef.data.16733224<br>https://esapubs.org/archive/
Terrestrial biome and ecoregion polygons	Olson et al.[42]	https://www.sciencebase.gov/catalog/item/<br>508fece8e4b0a1b43c29ca22

RESOURCE AVAILABILITY

Lead contact

Further information and requests for resources should be directed to and will be fulfilled by the lead contact, Emma Hughes (echughes8@gmail.com).

Materials availability

This study did not generate new unique reagents.

Data and code availability

Original datasets and code supporting the results are available in the University of Sheffield's ORDA repository, provided by figshare: https://doi.org/10.15131/shef.data.20004806.v1.

EXPERIMENTAL MODEL AND SUBJECT DETAILS

Morphological trait space

We used a raw dataset of ecologically relevant morphological traits from Hughes et al. 2022[9] for 8455 of 9993 bird species. Our selected traits include the main seven principal components of beak shape (accounting for 98.9% of the total variation in beak shape) and bill size (centroid size) derived from 3D scans of museum specimens,[9,17,35] and tarsus length (mm) and wing length (mm) taken from the corresponding museum specimens.[9] In addition, body size (g) was taken from the EltonTraits database.[66] These types of morphological traits have been closely linked to avian dietary and foraging ecology.[16,18] Bill size, wing length, tarsus length and body size were log10-transformed, and all trait data were then centred and re-scaled by standardising each to a mean of zero and unit variance (z-transformation). Finally, a principal components analysis (PCA) was run on the traits, and we selected the first eight PC axes (96.1% of total variation) from the resultant morphospace for analysis. Loadings for each individual trait on each principal component are provided in Table S2.

Threat status

We used data from the IUCN Red List,[19] to obtain threat statuses for each species with complete trait data (n = 8489), following the BirdTree[65] taxonomy used in our dataset. Species categorised as Data Deficient (DD) (n = 20), Extinct in the Wild (EW)/ Extinct (EX) (n = 4) or Critically Endangered (Possibly Extinct) (CR(PE)) (n = 9) were excluded from our dataset. Where a species under the BirdTree taxonomy was listed as multiple species in the IUCN Red List taxonomy, we assigned the mean categorisation value. The resultant dataset contained 8455 species, with 6731 categorised as Least Concern (LC), 812 as Near Threatened (NT), 527 as Vulnerable (VU), 274 as Endangered (EN), and 111 as Critically Endangered (CE).

Species pools

We defined a global pool of 8455 extant species with complete trait and threat status data. To account for regional and local spatial scales, we also generated species pools for 14 biomes and 814 ecoregions,[42] excluding “Lake” and “Rock and Ice” categorisations. To do this, we obtained global breeding and resident distribution maps for all extant and probably extant species in our dataset from BirdLife International,[43] and projected these, as well as a spatial layer of ecoregions, onto a 100 km x 100 km equal area grid under Behrman cylindrical equal-area projection. Next, we recorded the presence/ absence of each species, and the dominant ecoregion in each grid cell. As each ecoregion exists in only one biome, we further matched biome identity to each grid cell. All 8384 species across 820 ecoregions and 14 biomes were categorised in this way, and for each ecoregion and biome we extracted a species list. Forty-two species that were not categorised during this process as a result of very small distributions, were manually assigned to the correct biomes and ecoregions. Due to the dimensionality of the trait data, at least nine species are needed for trait space calculations and thus six ecoregions with fewer than nine species were removed from our dataset. Three species were found exclusively in one of the removed ecoregions, and these were also dropped from our ecoregion species pools. Therefore, our final fourteen biome and 814 ecoregion species pools comprised 8426 and 8423 of 9993 (84.3%) species, respectively, with complete trait, conservation status, and range data present.

METHOD DETAILS

Avian morphological and phylogenetic diversity measures

Our analyses were carried out at a global scale (across all 8455 bird species), regional scales (within biomes), and local scales (within ecoregions).

For each species pool, we first calculated the mean distance to centroid (i.e., the mean Euclidean distance from the morphospace centroid, also known as Functional Dispersion[20]), as a measure of morphospace size using the *dispaRity* R package (version 1.6.0).[67] Next, we sequentially dropped species from the most to least threatened IUCN category (CR > EN > VU > NT) and re-calculated the mean distance to centroid for the remaining species. Our focus was to examine changes in morphospace size as threatened species were lost from their respective pools. A reduction in morphospace size (i.e., a lower mean distance to centroid) is indicative of morphological homogenisation as species with more unique trait combinations than average are lost. We note that increases in mean distance to centroid can occur where species are primarily lost from the centre of morphospace. In addition, species could be lost such that no change in mean distance to centroid occurs. We therefore stress that this should not be used as evidence that species loss in these areas is not of conservation concern. Identifying significant incidences of morphological diversity loss is of crucial importance, alongside species loss, as the ecological consequences of morphological homogenisation are a particular conservation concern.

To account for phylogenetic uncertainty, we calculated phylogenetic diversity[3] on all 200 phylogenetic trees[65] for each species pool using the function *pd.query* in the R package *PhyloMeasures* (version 2.1).[68] Phylogenetic diversity calculations were repeated for each species pool after sequentially dropping species from each IUCN category (CR, EN, VU, NT).

QUANTIFICATION AND STATISTICAL ANALYSIS

All data quantification, analysis and visualisation were conducted in RStudio[64] version 1.4.1717 and R[63] version 4.1.1.

Phylogenetic signal across morphological traits

To assess the potential for decoupling of morphological diversity from phylogenetic history, we tested for multivariate phylogenetic signal across our morphological traits. We downloaded 200 complete species-level phylogenetic trees based on the Hackett backbone[69] from http://birdtree.org/ and[65] pruned each so that it only consisted of species in our dataset. We then used the transformPhylo.ML function in the R package MOTMOT (version 2.1.3)[70] to calculate the multivariate phylogenetic signal (Pagels λ (lambda)[71,72]) of our eight PCs across every tree (n=200) (See results and discussion and Figure S1). A value of 1 shows high and a value of 0 shows no phylogenetic signal in traits.

Simulating the impact of threatened species loss on morphological and phylogenetic diversity

As morphological and phylogenetic diversity correlate with species richness,[9,25,26] we constructed null models to test whether the species remaining after losing each IUCN category had mean distance to centroid and phylogenetic diversity values that deviated from expected given the observed species richness. To do this, we sampled 1000 null assemblages for each value of species richness after losing CR, EN, VU, and finally NT species. For the global analysis, species sampled could be from the whole avian class; for each biome, species could be drawn from that focal biome species pool; and for each ecoregion, species were sampled from that focal ecoregion pool. For each of the 1000 null assemblages, we calculated the mean distance to centroid, before calculating the mean and standard deviation of these 1000 values. Next, we calculated the standard effect size (SES) for each global (Figure 2), biome (Figure 3), and ecoregion (Figure 4) community, by taking the null mean distance to centroid from the observed mean distance to centroid and dividing by the standard deviation of the null values:

$$SES = \frac{observed - mean(null)}{sd(null)}$$

We followed the same protocol to calculate the SES for phylogenetic diversity. SES scores were calculated for each of the phylogenetic trees (n=200),[65] and we took the average SES score for each global (Figure 2), biome (Figure 3), and ecoregion (Figure 4) community after losing each IUCN threat category. A positive SES value indicates a higher mean distance to centroid or phylogenetic diversity value than expected, whereas a negative SES indicates a lower value. Exceptional values of mean distance to centroid and phylogenetic diversity were those that showed statistically significant deviation from expected (+/- 2), with exceptionally negative values (< -2) indicating morphological or phylogenetic homogenisation of communities above that expected from species loss alone.

Current Biology, Volume *32*

Supplemental Information

The homogenization of avian morphological and phylogenetic diversity under the global extinction crisis

Emma C. Hughes, David P. Edwards, and Gavin H. Thomas

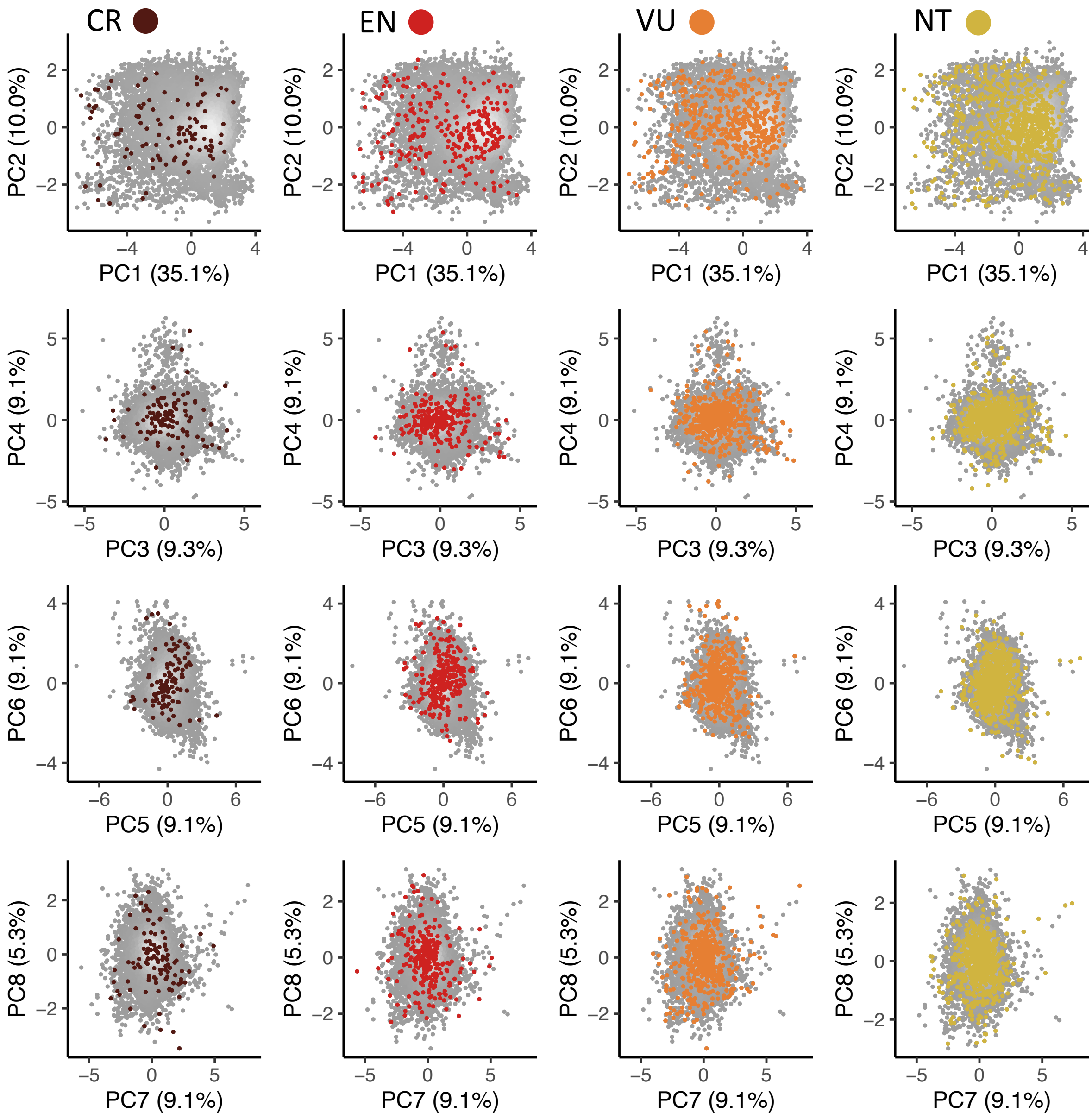

Figure S1: Scatterplots showing the first eight principal components of morphological traits, and the proportion of variation represented by each for each IUCN threat category. Related to Figure 1 and Table S1.

Species classified in the IUCN red list as Critically Endangered (CR), Endangered (EN), Vulnerable (VU), and Near Threatened (NT) are coloured dark red through to yellow, whilst all other species are grey. Light grey shows where species density is highest. The phylogenetic signal (mean λ) for each principal component is as follows: PC1 = 0.962, PC2 = 0.956, PC3 = 0.848, PC4 = 0.885, PC5 = 0.908, PC6 = 0.856, PC7 = 0.873, PC8 = 0.900.

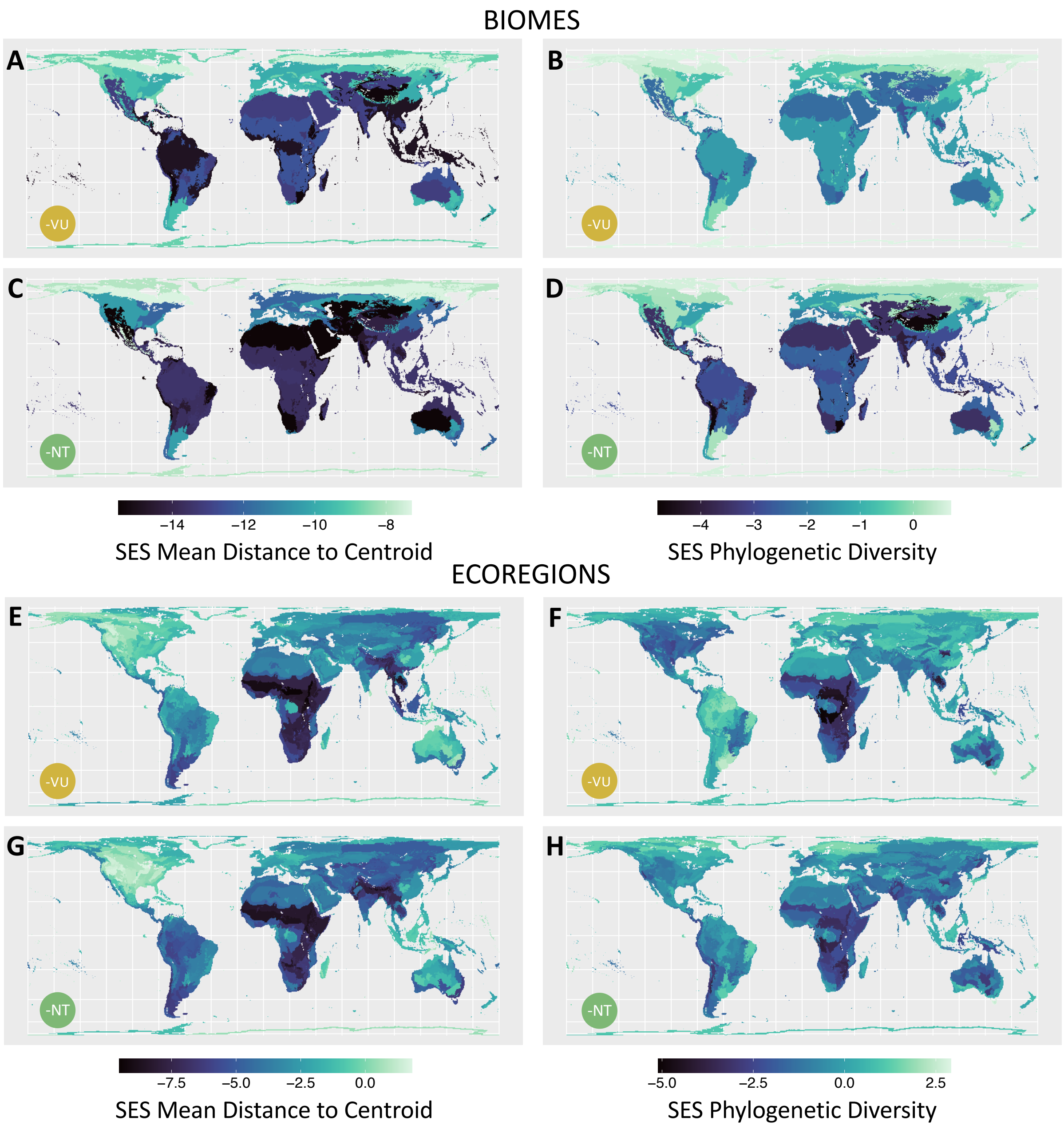

Figure S2: **Variation in morphological diversity and phylogenetic diversity across avian assemblages in each terrestrial biome and ecoregion. Related to Figures 3, 4, S3 and S4.**

Standard effect sizes (SES) for **A**) morphological and **B**) phylogenetic diversity were calculated after Critically Endangered, Endangered and Vulnerable species (-VU), and (**C, D**) additionally, when Near Threatened species (-NT) are dropped from 1000 simulated communities across 14 terrestrial biomes, and respectively across 814 terrestrial ecoregions (**E-H**). The darkest blue colour indicates where SES values are more negative than expected, with values < -2 showing significant homogenisation.

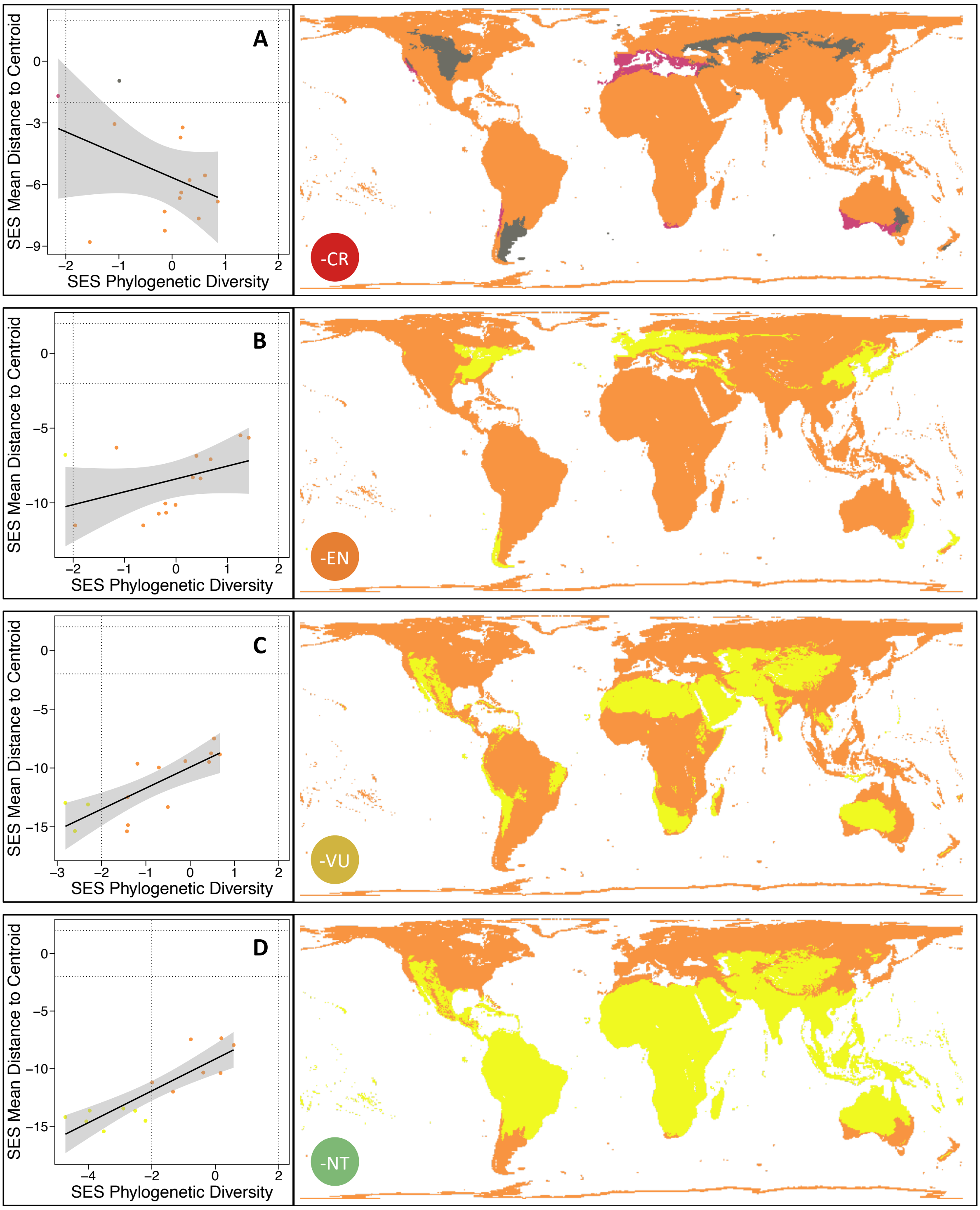

Figure S3: **Significant deviations from expected morphological and phylogenetic diversity across each terrestrial biome. Related to Figures 3 and S2.**
Standard effect sizes (SES) of morphological diversity (mean distance to centroid) and phylogenetic diversity of species assemblages in each global terrestrial biome (n = 14) where significant deviation from expected (+/- 2) is present. Homogenisation is indicated where SES is more negative than -2. Significant SES scores calculated when assemblages are missing **A)** Critically Endangered (-CR), plus **B)** Endangered (-EN), plus **C)** Vulnerable (-VU), plus **D)** Near Threatened (-NT) species. Dark grey indicates no significant deviation from expected.

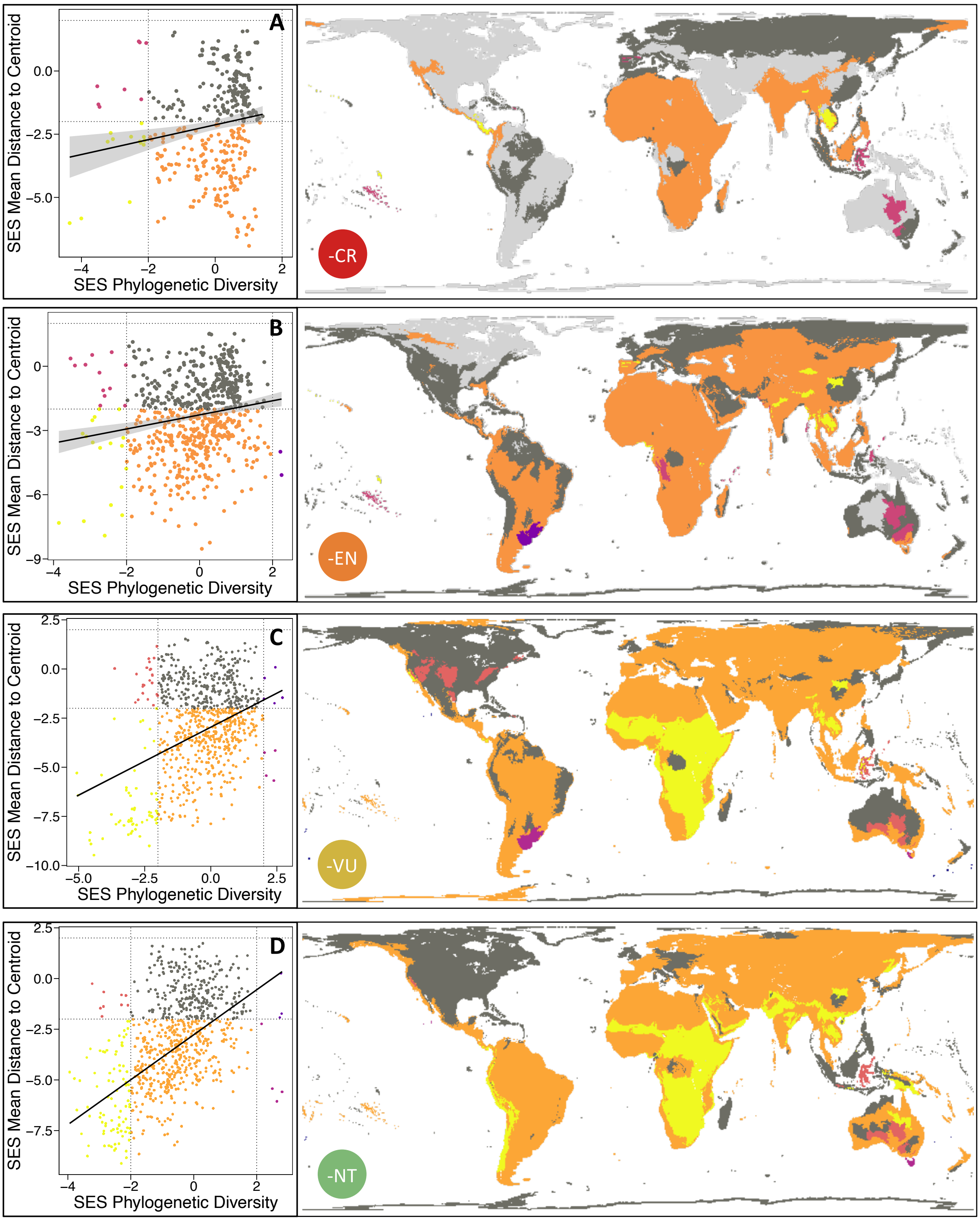

Figure S4: **Significant deviations from expected morphological and phylogenetic diversity across each terrestrial ecoregion. Related to Figures 4 and S2.**

Standard effect sizes (SES) of morphological diversity (mean distance to centroid) and phylogenetic diversity of species assemblages in each global terrestrial ecoregion (n = 814) where significant deviation from expected (+/- 2) is present. Homogenisation is indicated where SES is more negative than -2. Significant SES scores calculated when assemblages are missing **A)** Critically Endangered (-CR), plus **B)** Endangered (-EN), plus **C)** Vulnerable (-VU), plus **D)** Near Threatened (-NT) species. Dark grey indicates no significant deviation from expected. Light grey indicates ecoregions with no CR or EN species.

Table S1: Species richness, mean distance to centroid and the mean, standard deviation of null simulations used to calculate the SES. Related to Figure 1 and S1.

	Species richness	Mean distance to centroid	Null mean distance to centroid	Null SD distance to centroid	SES mean distance to centroid
**PC1**					
All species retained	8455	1.572	NA	NA	0
CR lost	8344	1.559	1.572	0.002	-7.732
EN lost	8070	1.533	1.572	0.003	-12.387
VU lost	7543	1.488	1.572	0.005	-16.320
NT lost (LC only retained)	6731	1.455	1.572	0.007	-15.768
**PC2**					
All species retained	8455	0.822	NA	NA	0
CR lost	8344	0.821	0.822	0.001	-0.323
EN lost	8070	0.819	0.822	0.001	-1.350
VU lost	7543	0.818	0.822	0.002	-1.373
NT lost (LC only retained)	6731	0.813	0.821	0.004	-2.275
**PC3**					
All species retained	8455	0.771	NA	NA	0
CR lost	8344	0.768	0.771	0.001	-3.541
EN lost	8070	0.760	0.771	0.002	-6.585
VU lost	7543	0.745	0.771	0.003	-9.920
NT lost (LC only retained)	6731	0.734	0.771	0.004	-10.443
**PC4**					
All species retained	8455	0.705	NA	NA	0
CR lost	8344	0.702	0.705	0.001	-2.480
EN lost	8070	0.702	0.705	0.002	-1.786
VU lost	7543	0.695	0.705	0.003	-3.724
NT lost (LC only retained)	6731	0.693	0.705	0.004	-2.997
**PC5**					
All species retained	8455	0.754	NA	NA	0
CR lost	8344	0.752	0.754	0.001	-1.961
EN lost	8070	0.747	0.754	0.002	-4.173
VU lost	7543	0.740	0.754	0.002	-5.815
NT lost (LC only retained)	6731	0.726	0.753	0.004	-7.703
**PC6**					
All species retained	8455	0.775	NA	NA	0
CR lost	8344	0.773	0.775	0.001	-2.634
EN lost	8070	0.770	0.775	0.002	-3.445

VU lost	7543	0.763	0.775	0.002	-5.083
NT lost (LC only retained)	6731	0.761	0.775	0.004	-4.007
**PC7**					
All species retained	8455	0.714	NA	NA	0
CR lost	8344	0.709	0.714	0.001	-5.419
EN lost	8070	0.702	0.714	0.002	-7.072
VU lost	7543	0.687	0.714	0.003	-10.732
NT lost (LC only retained)	6731	0.677	0.714	0.004	-9.615
**PC8**					
All species retained	8455	0.580	NA	NA	0
CR lost	8344	0.576	0.580	0.001	-5.061
EN lost	8070	0.573	0.580	0.001	-5.514
VU lost	7543	0.565	0.579	0.002	-7.317
NT lost (LC only retained)	6731	0.560	0.579	0.003	-6.721
**PC1-8**					
All species retained	8455	2.949	NA	NA	0
CR lost	8344	2.934	2.949	0.002	-7.887
EN lost	8070	2.906	2.949	0.004	-12.004
VU lost	7543	2.855	2.949	0.006	-15.937
NT lost (LC only retained)	6731	2.816	2.948	0.008	-15.796
**PC2-8**					
All species retained	8455	2.339	NA	NA	0
CR lost	8344	2.332	2.340	0.001	-5.243
EN lost	8070	2.318	2.340	0.003	-7.611
VU lost	7543	2.291	2.340	0.004	-11.333
NT lost (LC only retained)	6731	2.270	2.339	0.006	-10.681

Species richness, mean distance to centroid values (the average across all individual species Euclidean distance to the centroid of morphospace where Critically Endangered (CR), Endangered (EN), Vulnerable (VU) and Near Threatened (NT) species are sequentially lost), null mean distance to centroid (the average across all individual species in a simulated community where CR, EN, VU and NT species are sequentially lost), null standard deviation (SD) of the distance to centroid values (for each simulated community), and the standard effect size (SES) mean distance to centroid value ((mean distance to centroid – null mean distance to centroid)/null SD distance to centroid). SES values are significant +/- 2. Each null value was calculated from 1000 simulated communities. These calculations were repeated individually for PC1, PC2, PC3, PC4, PC5, PC6, PC7 and PC8, across all PCs (1-8), and PCs 2-8.

Table S2: Loadings for each individual trait on each principal component. Related to Figure S1 and Star Methods.

	PC1	PC2	PC3	PC4	PC5	PC6	PC7	PC8
Beak PC1	0.002	0.936	0.014	0.028	0.113	0.074	0.056	0.006
Beak PC2	-0.046	-0.084	0.225	0.274	0.163	0.901	-0.100	-0.104
Beak PC3	0.280	-0.019	0.306	0.461	0.143	-0.156	-0.142	0.739
Beak PC4	0.100	-0.044	0.450	-0.674	0.199	0.137	0.440	0.270
Beak PC5	-0.183	-0.046	0.476	0.354	0.435	-0.353	0.274	-0.442
Beak PC6	-0.044	0.083	0.532	0.088	-0.824	-0.018	0.034	-0.085
Beak PC7	0.051	-0.041	-0.337	0.344	-0.178	0.121	0.835	0.119
Centroid Size	-0.451	-0.279	0.026	0.006	<0.001	<0.001	<0.001	0.139
Body Mass	-0.493	0.113	0.006	0.053	<0.001	<0.001	<0.001	0.123
Tarsus Length	-0.438	0.043	-0.144	-0.026	<0.001	<0.001	<0.001	0.316
Wing Length	-0.483	0.103	0.087	-0.039	<0.001	<0.001	<0.001	0.140
